# Biosurfactants: Multifunctional Biomolecules of the 21st Century

**DOI:** 10.3390/ijms17030401

**Published:** 2016-03-18

**Authors:** Danyelle Khadydja F. Santos, Raquel D. Rufino, Juliana M. Luna, Valdemir A. Santos, Leonie A. Sarubbo

**Affiliations:** 1 Northeast Botechnology Network (RENORBIO), Federal Rural University of Pernambuco, Rua Dom Manoel de Medeiros, s/n, Dois Irmãos, 52171-900 Recife-PE, Brazil; danykhadydja@hotmail.com; 2 Center of Sciences and Technology, Catholic University of Pernambuco (UNICAP), Rua do Príncipe, 526, Boa Vista, 50050-900 Recife-PE, Brazil; raqueldrufino@yahoo.com.br (R.D.R.); julianamouraluna@gmail.com (J.M.L.); valdemir.alexandre@hotmail.com (V.A.S.); 3 Advanced Institute of Technology and Innovation (IATI), Rua Carlos Porto Carreiro, 70, Derby, 50070-090 Recife-PE, Brazil

**Keywords:** biosurfactant, surface tension, critical micelle concentration, biodegradability, functional properties, physiology, kinetics, recovery, industrial applications

## Abstract

In the era of global industrialisation, the exploration of natural resources has served as a source of experimentation for science and advanced technologies, giving rise to the manufacturing of products with high aggregate value in the world market, such as biosurfactants. Biosurfactants are amphiphilic microbial molecules with hydrophilic and hydrophobic moieties that partition at liquid/liquid, liquid/gas or liquid/solid interfaces. Such characteristics allow these biomolecules to play a key role in emulsification, foam formation, detergency and dispersal, which are desirable qualities in different industries. Biosurfactant production is considered one of the key technologies for development in the 21st century. Besides exerting a strong positive impact on the main global problems, biosurfactant production has considerable importance to the implantation of sustainable industrial processes, such as the use of renewable resources and “green” products. Biodegradability and low toxicity have led to the intensification of scientific studies on a wide range of industrial applications for biosurfactants in the field of bioremediation as well as the petroleum, food processing, health, chemical, agricultural and cosmetic industries. In this paper, we offer an extensive review regarding knowledge accumulated over the years and advances achieved in the incorporation of biomolecules in different industries.

## 1. Introduction

**Figure 1:**
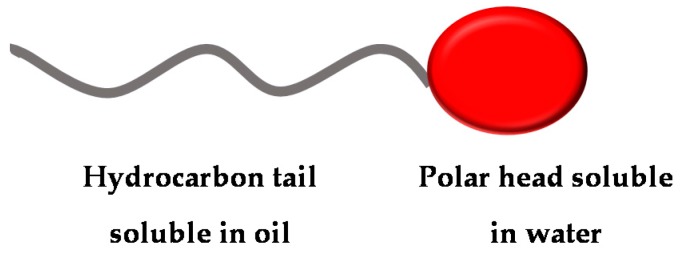
Surfactant molecule with apolar (hydrophobic) and polar (hydrophilic) moieties.

Surfactants are amphipathic compounds with both hydrophilic and hydrophobic moieties that preferentially partition between liquid interfaces with different degrees of polarity and hydrogen bridges, such as oil/water or air/water interfaces. The apolar moiety is often a hydrocarbon chain, whereas the polar moiety may be ionic (cationic or anionic), non-ionic or amphoteric [1,2], as illustrated in Figure 1.

**Figure 2:**
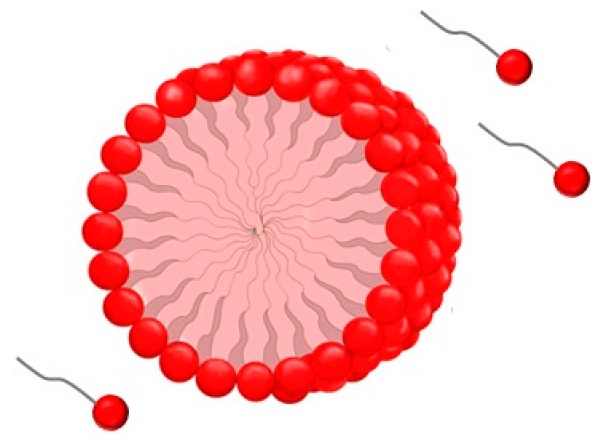
Schematic illustration of tensioactive agent and micelle formation.

**Figure 3:**
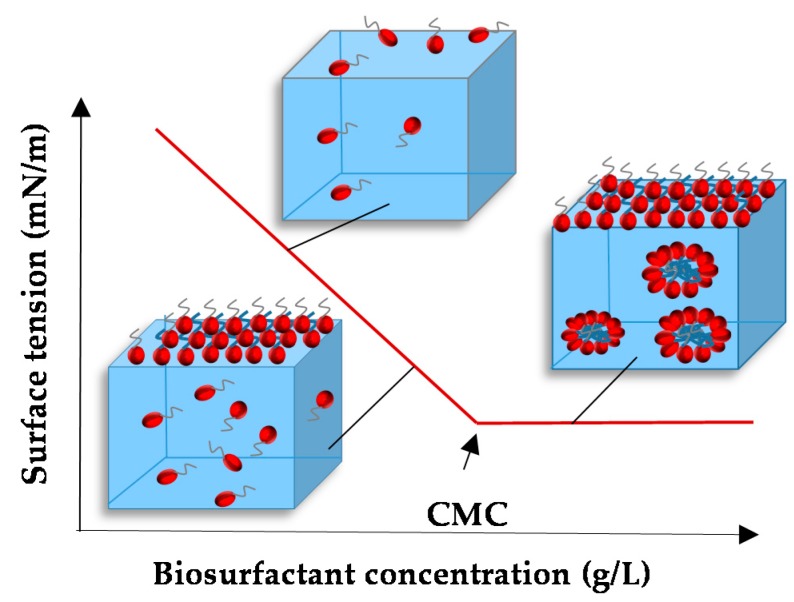
Illustration of regions in which micelle formation occurs (critical micelle concentration CMC).

Surfactants increase the solubility of hydrophilic molecules, thereby reducing both surface and interfacial tensions at the oil/water interface [3]. The critical micelle concentration (CMC) is the concentration of surfactant at which organised molecular assemblies, known as micelles, are formed (Figure 2) and corresponds to the point at which the tensioactive agent achieves the lowest stable surface tension (Figure 3) [4].

Most currently produced surfactants are chemically derived from petroleum. However, such synthetic tensioactive agents are generally toxic and difficult to break down through the action of microorganisms. In recent years, such problems have motivated the scientific community to seek surfactants that are more environmentally friendly, such as those achieved through microbial production, known as biosurfactants [5]. Moreover, concerns regarding the environment on the part of consumers and new environmental control legislation have led to the development of natural surfactants as an alternative to existing products.

Studies involving biosurfactants began in the 1960s and the use of these compounds has expanded in recent decades [2,6]. Biosurfactants have drawn the interest of different industries due to advantages such as structural diversity, low toxicity, greater biodegradability, ability to function in wide ranges of pH, temperature and salinity as well as greater selectivity, lower CMC and production involving renewable sources/industrial waste and industrial by-products [7,8,9]. The present review demonstrates the reasons for which biosurfactants are considered the multifunctional materials of the 21st century, with a description of concepts, properties, classification, modes of production, physiology and uses in the most diverse industries.

## 2. Producing Microorganisms

Microorganisms use a set of carbon sources and energy for growth. The combination of carbon sources with insoluble substrates facilitates the intracellular diffusion and production of different substances [10,11,12]. Microorganisms (yeasts, bacteria and some filamentous fungi) are capable of producing biosurfactants with different molecular structures and surface activities [4]. In recent decades, there has been an increase in scientific interest regarding the isolation of microorganisms that produce tensioactive molecules with good surfactant characteristics, such as a low CMC, low toxicity and high emulsifying activity [2].

The literature describes bacteria of the genera *Pseudomonas* and *Bacillus* as great biosurfactant producers [2]. However, most biosurfactants of a bacterial origin are inadequate for use in the food industry due to their possible pathogenic nature [13]. *Candida bombicola* and *Candida lipolytica* are among the most commonly studied yeasts for the production of biosurfactants. A key advantage of using yeasts, such as *Yarrowia lipolytica*, *Saccharomyces cerevisiae* and *Kluyveromyces lactis,* resides in their “generally regarded as safe” (GRAS) status. Organisms with GRAS status do not offer the risks of toxicity or pathogenicity, which allows their use in the food and pharmaceutical industries [4]. Table 1 displays a list of microorganisms that produce biosurfactants.

## 3. Classification

Most biosurfactants are either anionic or neutral, whereas those that contain amine groups are cationic. The hydrophobic moiety has long-chain fatty acids and the hydrophilic moiety can be a carbohydrate, cyclic peptide, amino acid, phosphate carboxyl acid or alcohol. The molar mass of biosurfactants generally ranges from 500 to 1500 Da [14]. Biosurfactants are generally categorised by their microbial origin and chemical composition, as follows [3,5,15].

### 3.1. Glycolipids

Rhamnolipids, sophorolipids and trehalolipids are the best known glycolipids [16]. Rhamnolipids were found as exoproducts of the pathogen *P. aeruginosa* and are a combination of α-l-rhamnopyranosyl-α-l-rhamnopyranosyl-β-hydroxydecanoyl-β-hydroxydecanoate (Rha-Rha-C10-C10) and α-l-rhamnopyranosyl-α-l-rhamnopyranosyl-β-hydroxydecanoate (Rha-Rha-C10) as well as their mono-rhamnolipid congeners (Rha-C10-C10 and Rha-C10) [17]. Sensitive analytical techniques have led to the discovery of rhamnolipid congeners and homologues (approximately 60) produced at different concentrations by species of *Pseudomonas* and bacteria belonging to other families, classes or even phyla [16]. For instance, various species of *Burkholderia* have been shown to produce rhamnolipids that have longer alkyl chains than those produced by *P. aeruginosa* [17,18,19]. Surface tensions values of 29 mN/m constitute a characteristic of such components, which can be produced using different substrates, such as alkanes, pyruvate, citrates, fructose, glycerol, olive oil and glucose [20]. Most studies involving rhamnolipids focus mainly on assessing the biodegradation efficiency of petroleum hydrocarbons [21,22]. Although researchers have found increased dissipation of target contaminant upon the addition of rhamnolipids, a decrease in biodegradation efficiency or no effect following rhamnolipid supplementation have also been reported [16,20]. The presence of surfactant molecules may induce changes in the microbial community, which, in turn, correspond to different degradation patterns. Interestingly, although rhamnolipids are considered biodegradable, few reports have demonstrated that these substances can be co-degraded or solely utilised as a carbon and energy source by various monocultures [18]. Rhamnolipids are described as potentially toxic to natural vegetation [23], but have also been found to reduce the toxicity of specific compounds by increasing hydrocarbon solubilisation, thereby facilitating biodegradation [24,25].

Sophorolipids are produced by yeasts that belong to the genus *Candida* [26,27]. These glycolipids have a dimeric carbohydrate sophorose linked to a long-chain hydroxyl fatty acid through a glycosidic bond. Sophorolipids and lactone form a sophorolipid that is preferable in many applications [28,29]. *C. bombicola* stands out among the different types of yeasts used in the production of these biosurfactants. Surface tension values of approximately 33 mN/m and a reduction in the surface tension of *n*-hexadecane and water from 40 to 5 mN/m has been recorded for these agents [30]. Mannosylerythritol lipids (MEL), which are yeast glycolipids, are one of the most promising biosurfactants known and are abundantly produced from vegetable oils by *Pseudozyma* (previously *Candida*) *antarctica* [31]. Trehalolipids are produced by species of *Mycobacterium*, *Nocardia* and *Corynebacterium*. Trehalolipids from *Arthrobacter* spp. and *Rhodococcus erythropolis* are able to lower surface and interfacial tensions in culture broth to 25–40 and 1–5 mN/m, respectively [5].

### 3.2. Fatty Acids, Phospholipids and Neutral Lipids

Different bacteria and yeasts produce large amounts of fatty acids and phospholipid surfactants during growth on n-alkanes. Phosphatidyl ethanolamine-rich vesicles are produced from *Acinetobacter* spp. and form optically clear microemulsions of alkanes in water. These biosurfactants are essential to medical applications. According to Gautam and Tyagi [28], phospholipid protein complex deficiency is the major cause of respiratory failure in the children born prematurely. The authors also suggest that the isolation and cloning of genes involved in the production of surfactants can be used in fermentative processes [28].

### 3.3. Polymeric Biosurfactants

Emulsan, lipomanan, alasan, liposan and other polysaccharide protein complexes are the best-studied polymeric biosurfactants. Emulsan is an emulsifier for hydrocarbons in water at concentrations as low as 0.001% to 0.01% [31,32]. Liposan is an extracellular water soluble emulsifier synthesised by *C. lipolytica* and is made up of 83% carbohydrates and 17% proteins. Chakrabarti [33] discuss the application of liposan as an emulsifier in the food and cosmetic industries.

### 3.4. Particulate Biosurfactants

Particulate biosurfactants partition extracellular membrane vesicles to form a microemulsion that exerts an influence on alkane uptake in microbial cells. The *Acinetobacter* spp. has vesicles with a diameter of 20 to 50 nm and a buoyant density of 1.158 cubic gcm composed of proteins, phospholipids and lipo-polysaccharides [5,33].

**Figure 4:**
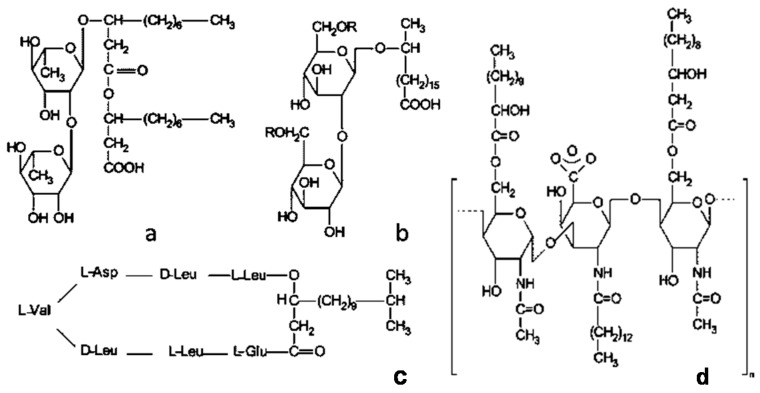
Chemical structure of most studied microbial surface-active compounds. (**a**) Rhamnolipid; (**b**) Sophorolipid; (**c**) Surfactin and (**d**) Emulsan.

Figure 4 illustrates the chemical structure of the most studied microbial surfactants. Table 1 also displays biosurfactant classes and their producers.

## 4. Properties

It is necessary to submit a biosurfactant to conservation methods to evaluate its properties (surface tension and dispersion) over a period of 120 days to estimate the commercial validity of the product. Thus, heating methods are used separately or in combination with potassium sorbate, which is a conservative that inhibits the growth of mould that is widely used in the production and conservation of foods. Some characteristics are common to the majority of biosurfactants and have advantages over conventional surfactants, as described below [5].

### 4.1. Surface and Interfacial Activity

Efficiency and effectiveness are essential characteristics of a good surfactant. Efficiency is measured by the CMC, whereas effectiveness is related to surface and interfacial tensions [34]. The CMC of biosurfactants ranges from 1 to 2000 mg/L, whereas interfacial (oil/water) and surface tensions are respectively approximately 1 and 30 mN/m. Good surfactants are able to reduce water surface tension from 72 to 35 mN/m and the interfacial tension of *n*-hexadecane from 40 to 1 mN/m.

### 4.2. Tolerance to Temperature, pH and Ionic Strength

Many biosurfactants can be used at high temperatures and pH values ranging from 2 to 12. Biosurfactants also tolerate a salt concentration up to 10%, whereas 2% NaCl is enough to inactivate synthetic surfactants.

### 4.3. Biodegradability

Biosurfactants are easily degraded by microorganisms in water and soil, making these compounds adequate for bioremediation and waste treatment.

### 4.4. Low Toxicity

Low degree of toxicity allows the use of biosurfactants in foods, cosmetics and pharmaceuticals. Low toxicity is also of fundamental importance to environmental applications.

Biosurfactants can be produced from largely available raw materials as well as industrial waste.

### 4.5. Specificity

Biosurfactants are complex molecules with specific functional groups and therefore often have specific action. This is of particular interest in the detoxification of different pollutants and the de-emulsification of industrial emulsions as well as specific food, pharmaceutical and cosmetic applications.

### 4.6. Biocompatibility and Digestibility

These properties allow the use of biomolecules in different industries, especially the food, pharmaceutical and cosmetic industries.

### 4.7. Emulsion Forming/Breaking

Biosurfactants can be either emulsifiers or de-emulsifiers. An emulsion is a heterogeneous system consisting of an immiscible liquid dispersed in another liquid in the form of droplets, the diameter of which generally exceeds 0.1 mm. There are two basic types of emulsion: oil-in-water (o/w) and water-in-oil (w/o). Emulsions have minimal stability, but the addition of biosurfactants can lead to an emulsion that remains stable for months or even years [35]. Liposan, which is a water-soluble emulsifier synthesised by *C. Lipolytica*, has been used with edible oils to form stable emulsions. Liposan is commonly used in the cosmetic and food industries for producing stable oil/water emulsions [4,36].

## 5. Factors Affecting Biosurfactant Production

The production of biosurfactants can be either spontaneous or induced by the presence of lipophilic compounds, variations in pH, temperature, aeration and agitation speed or when cell growth is maintained under conditions of stress, such as a low concentration of nitrogen [37]. The various physicochemical factors are discussed below [38].

### 5.1. Carbon Source

The carbon source plays an important role in the growth and production of biosurfactants by microorganisms and varies from species to species. A very low yield was found when only either glucose or vegetable oil was used for the production of a biosurfactant by *T. bombicola*, but the yield increased to 70 g/L when both carbon sources were provided together [39]. At a concentration of 80 and 40 g/L of glucose and soybean oil, respectively, the maximum yield of sophorose lipids was obtained by *T. bombicola* [40]. Even higher yields of sophorolipids (120 g/L) were produced with *C. bombicola* in eight days when sugar and oil were used as carbon sources [41]. When canola oil and glucose were used as carbon sources at concentrations of 10% each, maximum yield of sophorolipids (8 g/L) was obtained from *C. lipolytica* [42]. Moreover, when industrial waste was used for the production of a biosurfactant by *C. lipolytica*, the yield of the protein-lipid-carbohydrate complex was 4.5 g/L, with a reduction in the surface tension of distilled water from 71 to 32 mN/m [43]. A high production of bioemulsifier was obtained with *C. lipolytica* when supplemented with 1.5% glucose (*w*/*v*) [44]. *C. antarctica* and *C. apicola* yielded 13.4 and 7.3 g/L of sophorolipids, respectively, when soapstock was used at a concentration of 5% (*v*/*v*) [45]. The resting cells of *Pseudozyma (C. antarctica)* were found to covert C_12_ to C_18_ n-alkanes into mannosylerythritol lipids (MEL); the yield was 140 g/L after four weeks and the biosurfactant was able to emulsify soybean oil [46]. A change in the fatty acid constitution of the final biosurfactant occurred when the fatty acid composition was changed in the fermentation medium containing *C. ingens* [47].

### 5.2. Nitrogen Sources

This is the second most important supplement for the production of biosurfactants by microorganisms. In fermentative processes, the C/N ratio affects the buildup of metabolites. High C/N ratios (*i.e.*, low nitrogen levels) limit bacterial growth, favouring cell metabolism towards the production of metabolites. In constrast, excessive nitrogen leads to the synthesis of cellular material and limits the buildup of products [48]. Different organic and inorganic nitrogen sources have been used in the production of biosurfactants. Santa Anna *et al.* [49] describe the importance of nitrogen for the production of a biosurfactant by *P. aeruginosa* cultivated in a mineral medium containing 3% glycerol. As NaNO_3_ proved more effective than (NH_4_)_2_SO_4_, nutritional limitations clearly guide the cell metabolism to the formation of the product. Mulligan and Gibbs [50] report that *P. aeruginosa* uses nitrates, ammonium and amino acids as nitrogen sources. Nitrates are first reduced to nitrite and then ammonium. Ammonium is assimilated either by glutamate dehydrogenase (EC 1.4.1.4) to form glutamate or glutamine synthetase (EC 6.3.1.2) to form glutamine. Glutamine and α-ketoglutarate are then converted to glutamine by l-glutamine 2-oxoglutarate aminotransferase (EC 1.4.1.13). However, lipid formation rather than sugar is the rate-determining factor in the biosynthesis of rhamnolipids and nitrogen limitation can lead to the accumulation of lipids. In comparison to ammonium, the assimilation of nitrate is slower and simulates nitrogen limitation, which is favourable to the production of rhamnolipids. High yields of sophorose lipids, which are biosurfactants produced by the fungi *T. bombicola* and *C. Bombicola*, have been achieved using yeast extract and urea as the nitrogen source [51]. Moreover, high yields of mannosylerythritol lipid by *Candida* sp. SY16, *C. lipolytica* and *C. glabrata* have been achieved with ammonium nitrate and yeast extract [42,43,46,52,53,54].

### 5.3. Growth Conditions

Growth conditions (temperature, pH, agitation speed and oxygen) also influence biosurfactant production [37]. Species of the genus *Candida* produce maximum biosurfactant yields in a wide pH range, such as pH 5.7 for *C. glabrata* UCP 1002, pH 7.8 for *Candida* sp. SY16, pH 5.0 for *C. Lipolytica* and pH 6.0 for *C. batistae* [52,54,55,56]. Moreover, *Pichia anamola* and *Aspergillus ustus* produce maximum biosurfactant yield at pH 5.5 and 7.0, respectively [57,58]. Different microbial processes are affected by even a small change in temperature. The most favourable temperature for the production of biosurfactants by different fungi is 30 °C, as observed for different species of *Candida*, viz. *Candida* sp. SY16, *C. bombicola, C. batistae* and *T. bombicola* [39,51,52,56]. In case of *C. lipolytica*, 27 °C has been found to be the best temperature. Incubation time also exerts a significant effect on biosurfactant production. Microorganisms produce biosurfactants in different time intervals. Maximum biosurfactant production by *Aspergillus ustus* was found after five days of incubation, whereas the incubation periods for *C. bombicola* were seven, eight and 11 days [59,60]. Maximum biosurfactant production by *C. bombicola* grown in animal fat was found after 68 h of incubation [49]. Moreover, an increase in agitation speed favoured the accumulation of a biosurfactant by *P. aeruginosa* UCP 0992 grown in glycerol [61]. Oliveira *et al.* [62] studied the effect of a change in agitation speed of cultures from 50 to 200 rpm on *P. alcaligenes* cultivated in palm oil. The authors found that the increase in rotation velocity favoured a reduction in the surface tension of the cell-free broth to 27.6 mN/m. In contrast, Cunha *et al.* [63] found that agitation had a negative effect regarding a reduction in surface tension using a biosurfactant from *Serratia* sp. SVGG16 grown in a hydrocarbon culture.

## 6. Metabolic Pathways of Biosurfactant Production

**Figure 5:**
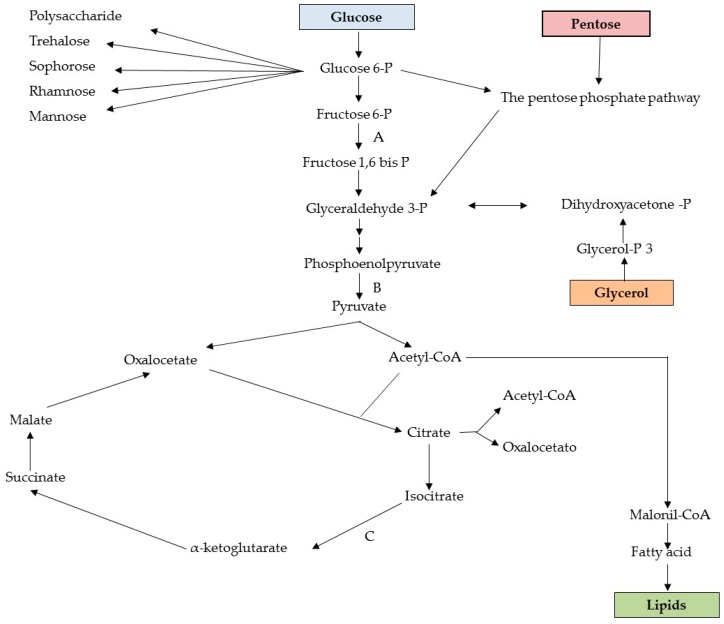
Intermediate metabolism related to synthesis of biosurfactant precursors with use of carbohydrates as substrate. Enzyme keys for control of carbon flow: (**A**) phosphofructokinase; (**B**) pyruvate kinase; (**C**) isocitrate dehydrogenase.

Hydrophilic substrates are primarily used by microorganisms for cell metabolism and the synthesis of the polar moiety of a biosurfactant, whereas hydrophobic substrates are used exclusively for the production of the hydrocarbon portion of the biosurfactant [37,64]. Diverse metabolic pathways are involved in the synthesis of precursors for biosurfactant production and depend on the nature of the main carbon sources employed in the culture medium. For instance, when carbohydrates are the only carbon source for the production of a glycolipid, the carbon flow is regulated in such as way that both lipogenic pathways (lipid formation) and the formation of the hydrophilic moiety through the glycolytic pathway are suppressed by the microbial metabolism, as illustrated in Figure 5 [65].

**Figure 6:**
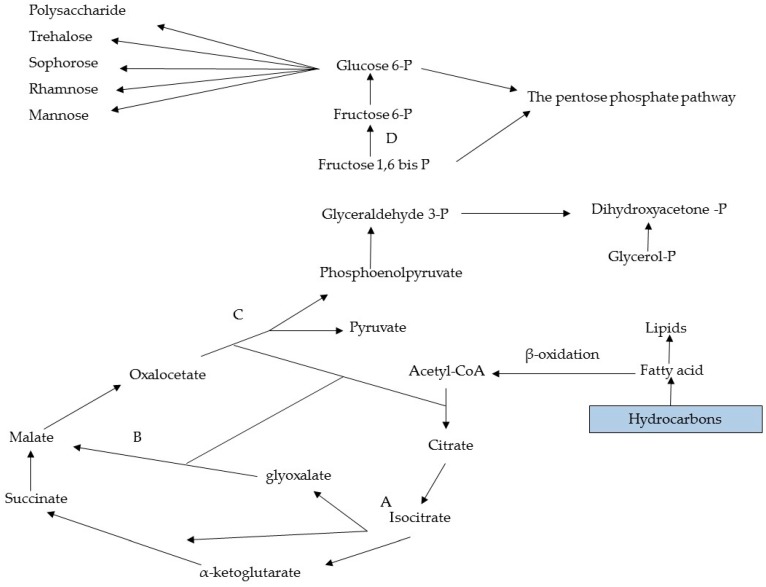
Intermediate metabolism related to synthesis of precursors of biosurfactant using hydrocarbons as substrate. Key enzymes: (**A**) isocitrate lyase; (**B**) malate synthase; (**C**) phosphoenolpyruvate; (**D**) fructose-1.

A hydrophilic substrate, such as glucose or glycerol, is degraded until forming intermediates of the glycolytic pathway, such as glucose 6-phosphate, which is one of the main precursors of carbohydrates found in the hydrophilic moiety of a biosurfactant. For the production of lipids, glucose is oxidised to pyruvate through glycolysis and pyruvate is then converted into acetyl-CoA, which produces malonyl-CoA when united with oxaloacetate, followed by conversion into a fatty acid, which is one of the precursors for the synthesis of lipids [66]. When a hydrocarbon is used as the carbon source, however, the microbial mechanism is mainly directed to the lipolytic pathway and gluconeogenesis (the formation of glucose through different hexose precursors), thereby allowing its use for the production of fatty acids or sugars. The gluconeogenesis pathway is activated for the production of sugars. This pathway consists of the oxidation of fatty acids through β-oxidation to acetyl-CoA (or propionyl-CoA in the case of odd chain fatty acids). Beginning with the formation of acetyl-CoA, the reactions involved in the synthesis of polysaccharide precursors, such as glucose 6-phosphate, are essentially the inverse of those involved in glycolysis. However, reactions catalysed by pyruvate kinase and phosphofructokinase-1 are irreversible. Thus, other enzymes exclusive to the process of gluconeogensis are required to avoid such reactions. Figure 6 illustrates the main reactions through to the formation of glucose 6-phosphate, which is the main precursor of polysaccharides and disaccharides formed for the production of the hydrophilic moiety of glycolipids [67].

According to Sydatk and Wagner [68], the biosynthesis of a surfactant occurs through four different routes: (a) carbohydrate and lipid synthesis; (b) synthesis of the carbohydrate half while the synthesis of the lipid half depends on the length of the chain of the carbon substrate in the medium; (c) synthesis of the lipid half while the synthesis of the carbon half depends on the substrate employed; and (d) synthesis of the carbon and lipid halves, which are both dependent on the substrate. Therefore, the length of the n-alkane chain used as the carbon source alters the biosynthesis of a surfactant. Kitamoto *et al.* [46] studied the production of manosilerythritol lipid (MEL) by the yeast *C. antarctica* in the presence of different n-alkanes and found that this species does not grow or produce a biosurfactant in media containing C_10_ to C_18_. However, production occurred when the species was grown in a medium containing C_12_ to C_18_ and octadekane as substrate led to the greatest yield. In contrast, production was minimal in media containing n-alkanes with more than 19 carbons.

## 7. Physiology

Biosurfactants are produced by microorganisms either through excretion or adhesion to cells, especially when cultivated on substrates that are insoluble in water. While the function in microbial cells is not yet fully understood, it has been speculated that biosurfactants are involved in the emulsification of insoluble substrates [37,69].

The main physiological role of biosurfactants is to allow microorganisms to grow on substrates that are insoluble in water through a reduction in surface tension between phases, making the substrate more available for uptake and metabolism. The uptake mechanisms of these substrates (such as, alkanes) are not yet fully clarified. The direct uptake of dissolved hydrocarbons in the aqueous phase, direct contact between cells and large hydrocarbon droplets, and the interaction with emulsified droplets (emulsion) have been described. Besides the emulsification of the carbon source, biosurfactants are also involved in the adhesion of microbial cells to hydrocarbons, as discussed in the following sections. Microorganism cell adsorption to insoluble substrates and the excretion of surfactant compounds allow growth on carbon sources [37].

## 8. Fermentation Kinetics

Biosurfactant production kinetics has considerable variation among different systems. For convenience, kinetic parameters are grouped as follows: (a) growth-associated production; (b) production under growth-limiting conditions; (c) production by resting or immobilised cells; and (d) production with precursor supplementation [37]. In growth-associated production, parallel relationships are found between growth, the use of the substrate and biosurfactant production. Production under growth-limiting conditions is characterised by an accentuated increase in biosurfactant concentration as a result of the limitation of one or more medium components. Production by resting or immobilised cells is a type of biosurfactant production in which there is no cell multiplication; the cells nonetheless continue to use the carbon source for biosurfactant synthesis. Investigators report that the addition of biosurfactant precursors to the medium leads to qualitative and quantitative changes in the final product.

## 9. Raw Materials for Biosurfactant Production

Current society is characterised by an increase in expenditures, the need to reuse materials and environmental concerns. Consequently, greater emphasis has been given to recovery, recycling and reuse. Indeed, the need for environmental preservation has led to the reuse of different industrial wastes. This is particularly valid for the food production industry, the waste products, effluents and by-products of which can be reused [70]. Industrial waste had piqued the interest of researchers as a low-cost substrate for biosurfactant production [71]. The selection of waste products should ensure the proper balance of nutrients to allow microbial growth and consequent biosurfactant production. Industrial waste with a high content of carbohydrates or lipids is ideal for use as substrate [71]. According to Barros *et al.* [34], the use of agro-industrial waste is one of the steps towards the implantation of feasible biosurfactant production on an industrial scale, for which the optimisation of the different variables involved is required.

The literature describes a number of waste products employed in biosurfactant production, such as vegetable oils, oily effluents [42,72,73], starchy effluents [74,75], animal fat [51,76,77,78], vegetable fat [79], vegetable cooking oil waste [72,80,81,82], soapstock [76,83,84] molasses [85,86,87,88], dairy industry waste (whey) [89], corn steep liquor [43,71,90,91,92], cassava flour wastewater [93], oil distillery waste [43,90,94,95] and glycerol [61]. Some of the most commonly employed industrial waste products for biosurfactant production are detailed below.

### 9.1. Olive Oil Mill Effluent (OOME)

Olive Oil Mill Effluent (OOME) is a concentrated black liquor with a water-soluble portion of ripe olives and water that is used for the extraction of olive oil. OOME has polyphenols that represent a challenge in terms of the environment disposal. However, it also contains nitrogen compounds (12 to 24 g/L), sugars (20 to 80 g/L), residual oil (0.3 to 5 g/L) and organic acids (5 to 15 g/L). Mercade *et al.* [73] successfully employed OOME for the strain *Pseudomonas* sp. to produce rhamnolipids.

### 9.2. Animal Fat

Animal fat and lard can be obtained in large quantities from the meat processing industry and have been used as a medium for cooking foods. Recently, however, such fats have lost a large part of the market to vegetable oils due to the lower degree of harm to health caused by the latter [70]. Animal fat stimulates the production of sophorolipids by the yeast *C. bombicola* [51]. Using animal fat and corn steep liquor, Santos *et al.* [77,78] achieved maximum glycolipid production by the yeast *C. lipolytica* UCP 0988. The authors also report that the product has uses in bioremediation as well as oil mobilisation and recovery.

### 9.3. Frying Oils

Fry oil and edible fats are considered great carbon sources for biosurfactant production. Vegetable oils constitute a lipid carbon source and are mainly comprised of saturated or unsaturated fatty acids with chains of 16 to 18 carbon atoms [9]. Different oils are adequate substrates for biosurfactants. Babassu oil (5% *v*/*v*) with a carbon source (1% glucose *w*/*v*) is a good medium for biosurfactant production. Sarubbo *et al.* [96] found that two strains of *C. lipolytica* (1055 and 1120) produce biosurfactants toward the final of the exponential growth phase and onset of the stationary phase. Sunflower and olive oils have proven to be adequate energy and carbon sources for the production of biosurfactants. *P. aeruginosa* strains produce a biosurfactant on residue from corn, soybean and canola oil plants [97,98]. Canola oil residue and sodium nitrate has been reported adequate for microbial growth and the production of up to 8.50 g/L of rhamnoipids. The combination of glucose and canola oil has been used for the successful production of a biosurfactant by *C. lipolytica* [42].

### 9.4. Soapstocks

Oil cakes or soapstocks are produced from oilseed processing involving the refining of seed-based edible oils with the use of chemicals [76]. Soapstock has been used together with sunflower oil, olive oil or soybean oil as substates to produce rhamnolipids. Yields as high as 15.9 g/L have been reported using *P. aeruginosa* in a soapstock medium [83]. Soapstock and oil refinery wastes have been used with *C. antarctica* or *C. apicola* for biosurfactant production, achieving a greater yield than that in the medium without oil refinery residue [45]. Shabtai [84] also report the production of two extracellular heteropolysaccharides (emulsan and biodispersan) by *A. calcoaceticus* and *A. calcoaceticus*, respectively, using soapstock as a carbon source.

### 9.5. Molasses

Molasses is a by-product of sugarcane and beet processing. This inexpensive substrate has dry matter (75%), non-sugar organic matter (9%–12%), protein (2.5%), and potassium (1.5%–5.0%), as well as magnesium, phosphorus and calcium (≈1%). The inositol, biotin, thiamine and pantothenic acid contents (1%–3%) give molasses its thick consistency and brown colour. The high sugar content (48%–56%) makes molasses adequate for biosurfactant production by different microorganisms. Laboratories have used molasses for the production of different microbial metabolites. According to Makkar and Cameotra [88], *Bacillus subtilis* in a minimal medium supplemented with molasses as the carbon source produces a biosurfactant. Joshi *et al.* [99] used molasses as well as other carbon sources to produce biosurfactants from strains of *Bacillus*.

### 9.6. Whey

The dairy industry produces large quantities of whey, such as whey waste, cheese whey, curd whey and lactic whey, all of which can be used as substrates for the microbial production of metabolites [70,100,101,102]. A high amount of lactose (approximately 75%) is found in lactic whey. Other components, such as proteins, vitamins and organic acids, are good sources for microbial growth and biosurfactant production [75]. Moreover, whey disposal represents a major pollution problem, especially in countries that depend on a dairy economy [103]. Thus, the disposal of this by-product represents a waste of a widely available substrate and an environmental hazard.

### 9.7. Corn Steep Liquor

The agro-industry of corn-based products through wet processing results in both solid and liquid by-products, which, when disposed improperly, become a source of contamination and harm to the environment. Corn steep liquor is a by-product of the washing water and soaking of kernels as well as fractioning into starch and germen (oil) that contains 40% solid matter. This by-product consists of 21% to 45% proteins, 20% to 26% lactic acid, approximately 8% ash (containing Ca^2+^, Mg^2+^, K^+^, *etc.*), approximately 3% carbohydrates and a low fat content (0.9% to 1.2%) [103,104,105]. Nut oil refinery residue and corn steep liquor are low-cost nutrients for the production of glycolipids by *C. sphaerica* (UCP 0995). The biosurfactant of this strain mobilises and removes up to 95% of motor oil on sand, making it useful for bioremediation [89,94,106]. Silva *et al.* [107] also report the production of a biosurfactant from *P. cepacia* grown in mineral medium supplemented with 2.0% corn steep liquor and 2.0% soybean waste frying oil.

### 9.8. Starchy Substrates

Abundant starch-based substrates also constitute renewable carbon sources. The potato processing industry produces significant amounts of starch-rich waste that are adequate for biosurfactant production. Besides the approximately 80% water content, potato waste has carbohydrates (17%), proteins (2%) and fats (0.1%) as well as inorganic minerals, trace elements and vitamins [103]. As an example, Fox and Bala investigated a commercially prepared potato starch in a mineral salt medium for the production of a biosurfactant by *B. subtilis* [74]. Cassava wastewater, which is another carbohydrate-rich waste product generated in large amounts, has been used for the production of surfactin by *B. Subtilis* 35. Other starchy wastes, such as rice water and cereal processing wastewater, have the potential to permit microbial growth and biosurfactant production [108].

## 10. Recovery of Biosurfactants

The production of low-coast biosurfactants is unlikely due to the complicated recovery process. Process development is conduted in order to obtain biosurfactants that can be recovered easily and inexpensively. In many biotechnological processes, downstream processing accounts for 70%–80% of production costs. For economic reasons, most biosurfactant production processes need to involve spent whole-cell culture broths or other crude preparations [11,103,109]. Extraction with chloroform-methanol, dichloromethane-methanol, butanol, ethyl acetate, pentane, hexane, acetic acid, ether, *etc.* constitutes the most commonly used method in biosurfactant downstream processing. The most widely employed products are different ratios of chloroform and methanol, which facilitate the adjustment of the polarity of the extraction agent to the extractable target material. The disadvantages of using organic solvents for biosurfactant recovery include the large amount of solvent required and the increase in production costs due to the price of expensive solvents. Chloroform is a toxic chloro-organic compound that is harmful to human health and the environment. Thus, there is a need for inexpensive solvents with low toxicity for biosurfactant extraction processes that are suitable for industrial applications. Other product precipitation techniques have also been reported, such as precipitation with ammonium sulphate, centrifugation and adsorption. Biosurfactant recovery depends mainly on the ionic charge, water solubility and location (intracellular, extracellular or cell bond) [103,109]. Foam fractionation is a solvent-free method that separates biosurfactant molecules adsorbed to air bubbles in the culture medium. Biosurfactant production involves continuous foam formation due to the high surface activity. Foam in the broth interferes with mass and heat transfer processes, thereby affecting productivity. However, foam is beneficial to biosurfactant production, as it assists in the continuous removal of product, and therefore production and recovery processes can be accomplished in a single stage [110]. Continuous foam fractionation in the fermentation process helps prevent the accumulation of product that could otherwise inhibit biomass growth and product formation and also facilitates extended biosurfactant production in fed-batch or continuous mode operations. Moreover, biosurfactants do not readily undergo denaturation due to their small size and simple structure [111].

More research and development are required to optimise existing recovery processes to make such processes both commercially viable and more competitive [39,103]. Table 2 lists the most common biosurfactant recovery techniques and their advantages.

## 11. Industrial Applications of Biosurfactants

Biosurfactants have a wide range of biotechnological applications in petroleum, foods, beverages, cosmetics, detergents, textiles, paints, mining, cellulose, pharmaceutics and nanotechnology [112]. Currently, the main market is the petroleum industry. Biosurfactants can be used for oil residue recovery from storage tanks, other oil recovery processes, the cleanup of oil spills and the bioremediation of both soil and water [2,113]. Table 3 offers a summary of the uses of biosurfactants in different industries. The main biotechnological applications are detailed in the following sections.

### 11.1. Petroleum Recovery

**Figure 7:**
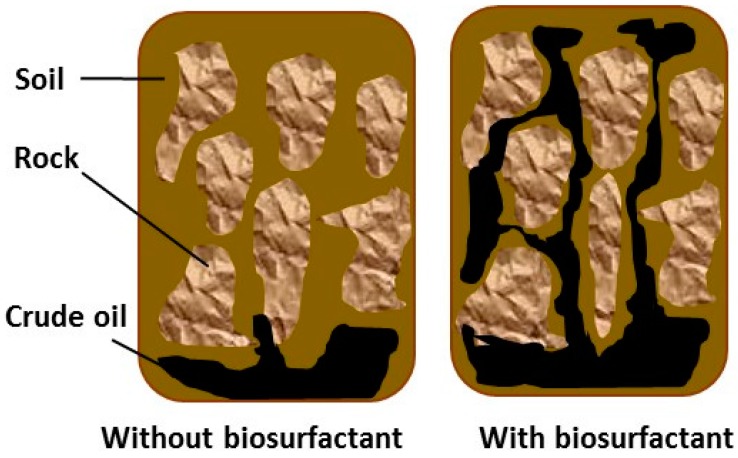
Enhanced oil recovery mechanism by biosurfactants.

Petroleum is an essential energy source and driving force of economic development. The US Department of Energy reports that fossil fuels constitute 83% of all primary energy sources in the country and petroleum accounts for 57% of such products. Indeed, 19.2 million cubic metres of petroleum were consumed per day in 2010 [114]. The USA produces 870,000 m^3^ of crude oil from 530 thousand production wells, 35% of which produce 0.16 m^3^/day and 79% produce < 1.59 m^3^/day [115]. Through oil recovery processes, these oil wells produce only one third to half of the petroleum originally present at the sites. Oil residue in small pores within petroleum reservoirs accounts for 50% to 65% of oil and is trapped by high forces of capillarity as well as interfacial tension between the hydrocarbon and aqueous phases. Different reductions in interfacial tension are needed for the mobilisation of this hydrocarbon [116,117], which is only achieved with the use of surfactant concentrations significantly higher than that required for the formation of micelles [118,119]. In enhanced oil recovery, the use of heat, tensioactive agents, microbial processes and gas injection leads to the recovery of a significant portion of the retained oil. However, the high cost of chemical tensioactive agents hinders widespread use of surfactants in oil recovery processes. Thus, biosurfactants have been employed to reduce the interfacial tension between oil/water and oil/rock, which leads to a reduction in the capillary forces that impede oil from moving through rock pores (Figure 7). Biosurfactants also form an emulsion at the oil-water interface, which stabilises the desorbed oil in water and allows oil removal along with the injection water [1,8].

### 11.2. Bioremediation

Oil spills occur during cargo transportation or in the form of industrial oil and by-product spills. Petroleum exerts a negative effect on cell membranes in living organisms, offering considerable risk of contamination to both marine and terrestrial ecosystems [2,114].

The US Environmental Protection Agency proposes different physical, chemical and biological technologies for the treatment of contaminated soil [126], one of the most studied of which is bioremediation. This process involves the natural degradation capacity of plants and microorganisms for either the partial conversion of contaminants into less toxic compounds or the complete conversion of such substances into carbon dioxide and water.

Larger degrading microorganism populations lead to a quicker, more efficient bioremediation processes. Therefore, this technique can be conducted through biostimulation, which consists of stimulating the growth of microorganisms present at the contaminated site. The process involves the introduction of specific electron receptors, oxygen and nutrients for the degradation of the contaminant as well as substances to correct the pH. Bioremediation can also be performed through bioaugmentation, in which indigenous (allochthonous) microorganisms are added to the contaminated environment to accelerate and complete the degradation of the pollutant [114].

Bioremediation played an important role in the cleanup of the 41 million litre oil spill caused by the oil tanker Exxon Valdez in the Gulf of Alaska in 1989, giving rise to the development of this technology and demonstrating that there are good reasons to believe in the effective application of this treatment method in future oil spills under the appropriate circumstances [114]. In the accident with the Exxon Valdez, the first measure taken was physical washing with high-pressure water. Chemical surfactants were then applied in polluted areas to accelerate the growth and activity of petroleum-degrading microorganisms. Two or three weeks later, the regions treated with surfactants were significantly cleaner than control areas. However, it was difficult to evaluate the exact effects of the treatment due to the heterogeneity of the contamination. Nonetheless, subsequent studies have demonstrated the importance of the use of surfactants to enhance the biodegradation of oil [114,127].

While bioremediation is an effective, environmentally friendly method, the time and costs involved make this process unviable for the treatment of large amounts of waste [128]. Thus, the use of biosurfactants emerges as a safe alternative for improving the solubility of hydrophobic compounds by allowing the desorption and solubilisation of hydrocarbons and facilitating the assimilation of these compounds by microbial cells [129].

**Figure 8:**
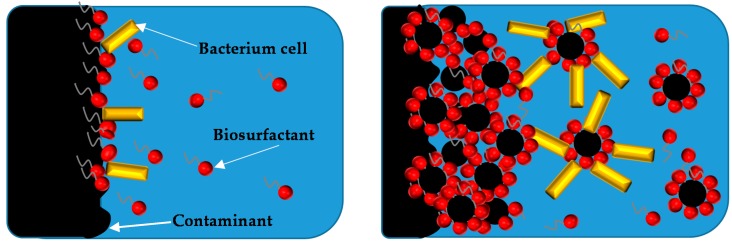
Illustration of biosurfactant action on petroleum.

The biodegradation of oil-derived hydrocarbons by biosurfactants occurs through two mechanisms. The first involves an increase in the bioavailability of the hydrophobic substrate to microorganisms, with a consequent reduction in surface tension of the medium around the bacterium as well as a reduction in interfacial tension between the cell wall and hydrocarbon molecules. The other mechanism involves the interaction between the biosurfactant and cell surface, leading to changes in the membrane, facilitating hydrocarbon adherence (increase in hydrophobicity) and reducing the lipopolysaccharide index of the cell wall without damaging the membrane. Thus, biosurfactants block the formation of hydrogen bridges and allow hydrophobic-hydrophilic interactions, which cause molecular rearrangements and reduce the surface tension of the liquid by increasing its surface area as well as promoting bioavailability and consequent biodegradability [130,131]. Figure 8 illustrates the action of biosurfactants in increasing the surface area of oil droplets as well as facilitating access to a greater number of bacteria and consequently producing a greater biomass.

### 11.3. Removal of Hydrophobic Organic Pollutants

The application of biosurfactants for the removal of contaminants from soil is less well known than the advanced application of these compounds in bioremediation processes, since removal efficiency is driven mainly by the physicochemical properties of the biosurfactant rather than the effects on metabolic activity or changes in the properties of the cell surface. However, the mechanisms that affect the mobilisation and solubilisation of hydrocarbons in soils are similar to those involved in the enhancement of bioavailability for bioremediation [131,132].

Biosurfactants enhance the removal of hydrocarbons through biodegradation, solubilisation, mobilisation or emulsification [8]. Solubilisation capacity depends on the ability of the surfactant to increase the solubility of hydrophobic components in the aqueous phase. A considerable increase in this capacity occurs above the CMC due to the partitioning of the hydrocarbon in the hydrophobic portion of the micelles. In this process, greater concentrations of surfactants are normally required, since the solubility of the hydrocarbon components in the solution depends wholly on the concentration of the surfactant [8]. Mobilisation occurs at concentrations below the CMC and is divided into displacement and dispersion. Displacement consists of the release of hydrocarbon droplets from the porous medium due to the reduction in interfacial tension. Using a theoretical explanation, hydrocarbon removal is possible when the interfacial tension between the aqueous and oil phases is sufficiently reduced to overcome the forces of capillarity that cause the formation of residual saturation. Dispersion is a process by which a hydrocarbon is dispersed in the aqueous phase as tiny emulsions. Emulsions are not generally thermodynamically stable, but may remain stable for significant periods of time due to kinetic restrictions. Dispersion is related to interfacial tension and surfactant concentration and differs from displacement, which is related only to interfacial tension between the aqueous and hydrophobic phases, with no formation of emulsion [132].

The efficiency of a surfactant in the removal of hydrophobic compounds also depends on the pH and ionic strength of the solution, which can alter the arrangement of the aggregated micelles and sorption of the surfactant to the soil, which, in turn, limits the transport of the hydrocarbon by the surfactant. Different biosurfactants have been tested for the removal of petroleum-derived products from contaminated soil and water. Rhaminolipids have been successfully used in biotechnological decontamination processes [2,7,17]. Other surfactants produced by species of *Pseudomonas* [110,133], *Bacillus* [5,134], and *Candida* [109,113,135,136,137], have also been successfully used in the remediation of soil.

### 11.4. Removal of Heavy Metals

Heavy metals and radionuclides are persistent soil contaminants. Increases in levels of heavy metals in soil have been reported in many industrialised countries. Metals and metalloids, such as chromium, cadmium, mercury and lead, can threaten ecosystems and human health through either the food chain or direct exposure to contaminated soil and water [1,12]. As different technologies can be used in combination for the treatment of organic pollutants and heavy metals, biosurfactants can be used in the removal of hydrophobic organic compounds and heavy metals [138,139,140]. Heavy metals mainly adsorb to the surface of soil in the form of ions or the precipitation of metal compounds. Unlike organic contaminants, heavy metals are removed from soil through surfactant-associated complexation [141] and ion exchange [142]. Therefore, surfactant-enhanced washing and surfactant-enhanced bio-extraction can be applied to the remediation of soils contaminated with heavy metals.

Surfactants in solutions facilitate the solubilisation, dispersion and desorption of contaminants and allow the reuse of the soil [143]. Decontamination tests have been performed with different synthetic surfactants [144,145], but the desire to replace such compounds with natural surfactants has led to research into the use of biosurfactants [119]. Studies have demonstrated the potential of surfactin, rhamnolipids and sophorolipids [146,147,148]. The ionic nature, biodegradability, low toxicity and excellent surface properties make biosurfactants adequate for the removal of heavy metals from sediment and soil. According to Mulligan [119], removal is possible with different concentrations of biosurfactants. Das *et al.* [149] found that the removal of cadmium using an aqueous solution also occurred at concentrations below the CMC, while a concentration fivefold greater than the CMC resulted in the virtually complete removal of 100 ppm of metal ions. Wen *et al.* [150] studied the degradation of a rhamnolipid in soils contaminated by cadmium and zinc and found that this compound could remain in the soil long enough to enhance the phytoextraction of the metals.

**Figure 9:**
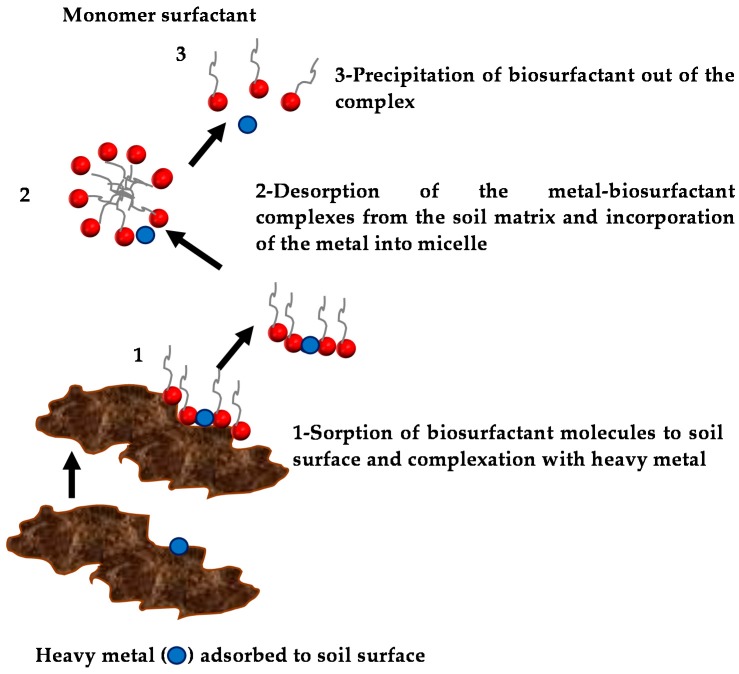
Mechanism of heavy metal removal by biosurfactants.

The removal of metals by ionic biosurfactants is thought to occur in the following order (Figure 9): (1) sorption of the biosurfactant to the soil surface and complexation with the metal; (2) detachment of the metal from the soil to the solution; and (3) association with micelles. Heavy metals are trapped within the micelles through electrostatic interactions and can be easily recovered through precipitation or membrane separation methods [151].

Anionic biosurfactants create non-ionic complexes with metals through ionic bonds. As such bonds are stronger than those between the metal and soil, the metal-biosurfactant complex is detached from the soil due to the reduction in interfacial tension. Cationic biosurfactants can replace similarly charged metal ions through ion exchange (competition for negatively charged surfaces). Surfactant micelles can also be used to remove metal ions from the soil surface [115].

Biosurfactants offer indisputable advantages, since surfactant-producing microorganisms do not need to survive in contaminated soil, although the continual addition of biosurfactant is required in the process [8]. Biosurfactants have also been applied in mining processes. Tensioactive compounds produced by *Pseudomonas* sp. and *Alcaligenes* sp. have been used for the floatation and separation of calcite and scheelite, with recovery rates of 95% for CaWO_4_ and 30% for CaCO_3_, whereas conventional chemical reagents are not capable of separating these two minerals [152]. Slizovskiy *et al.* [153] studied the enhanced remediation of soils contaminated with heavy metals using the cationic surfactant 1-dodecylpyridinium chloride (DPC), the non-ionic surfactant oleyl dimethyl benzyl ammonium chloride (trade name Ammonyx KP) and the ionic rhamnolipid biosurfactant (trade name JBR-425); the latter substance exhibited the best elution with regard to Zn (39%), Cu (56%), Pb (68%) and Cd (43%). Almeida *et al.* [154] studied the impact of surfactants on the removal of Cu by the salt marsh plant *Halimione portulacoides*. TX-100 and SDS (sodium dodecyl sulfate) were favourable to Cu harvesting and transportation in plant roots, but did not affect the transportation of Cu in the stem and leaves. The results of this study suggest that surfactants promote phytoremediation through a change in the membrane permeability of root cells. Therefore, surfactants can promote the desorption of metals and uptake by plants [1].

Rhamnolipids and soapberry-derived saponin have recently been found to assist in the removal of chromium and arsenic oxyanions from soils or the ore waste of mines [155,156]. Biosurfactants produced by yeast of the genus *Candida* have been successfully used in heavy metal flotation, demonstrating the ability to remove more than 90% of cations in columns and air-dissolved flotation processes [157,158]. *C. lipolytica* produces a biosurfactant that has also been used for the removal of heavy metals and petroleum byproducts using a soil barrier [90]. The biosurfactant significantly reduced soil permeability, thereby demonstrating its usefulness in reactive barriers, with the removal of approximately 96% of Zn and Cu as well as a reduction in Pb and Cd concentrations in groundwater.

### 11.5. Food Industry

Emulsification is important for the formation of consistency and texture in foods as well as phase dispersion and the solubilisation of aromas [4,159]. The general function of emulsifiers in food products is to stabilise the emulsion by controlling the agglomeration of fat globules and stabilising aerated systems [4,152]. An emulsion has at least one immiscible liquid (discontinuous internal phase) dispersed in another (continuous outer phase) in the form of droplets. The stability of this system is minimal, but can be enhanced by the addition of a surfactant, which reduces surface energy between the two phases by a reduction in interfacial tension, thereby preventing particle coalescence through the formation of steric and electrostatic barriers. Examples of processed foods that are emulsions include heavy cream, butter, mayonnaise, salad dressings, fillings, *etc.* [35]. Other uses for emulsifiers have been described, such as improving the texture and shelf life of products containing starch, the formation of complexes, altering the rheological properties of wheat flour and interactions with gluten as well as improving the consistency and texture of fat-based products through the control of polymorphism and the crystalline structure of fats [4].

Biosurfactants can also be used as emulsifiers in the processing of raw materials, the control of fat globule agglomeration, the stabilisation of aerated systems and an improvement in the consistency of fat-based products. The use of rhaminolipids to improve the emulsifying properties of frozen desserts, butter and croissants has also been reported [4,111]. For instance, *Candida utilis* produces a bioemulsifier used in processed salad dressings [160]. A manoprotein produced by *Saccharomyces cerevisiae* stabilises water/oil emulsions in cookies, mayonnaise and ice cream, *etc.* [13,161]. However, the food industry has not yet made widescale use of biosurfactants. Many of the properties of biosurfactants and their regulation as new ingredients for foods are pending approval.

### 11.6. Medicine

Biosurfactants have also been used in different biological (therapeutic) applications due to their fungicidal, bactericidal, insecticidal and anti-viral properties as well as use as anti-adhesive agents and enzyme inhibitors [111,116,117]. A number of rhaminolipids exhibit antibacterial activity. For instance, Abalos *et al.* [162] identified six rhaminolipids in cultures of *P. aeruginosa* AT10 grown on soybean oil refinery residue and evaluated the antimicrobial properties of the solution. These rhaminolipids exhibited excellent antifungal properties against different fungi at concentrations ranging from 16 to 32 µg/mL. *C. bombicola*-derived sophorolipids inhibited the growth of both Gram-negative and Gram-positive bacteria with a minimum inhibitory concentration of approximately 30 and 1 mg/mL in a contact time of 2 and 4 h, respectively, for *E. coli* (ATCC 8739) and *P. aeruginosa* (ATCC 9027) as well as 6 and 1 mg/mL in a contact time of 4 h for *S. aureus* (ATCC 6358) and *B. subtilis* (ATCC6633), respectively [163].

Despite the number of publications describing the antimicrobial activity of biosurfactants and patents related to their usage, real applications in the pharmaceutical, biomedical and health industries remains quite limited [164]. Some lipopeptides, such as daptomycin, have reached a commercial antibiotic status [165]. Daptomycin, is a branched cyclic lipopeptide isolated from *Streptomyces roseosporus* cultures and is produced by Cubist Pharmaceuticals under the name Cubicin^®^ [166]. This drug was approved in 2003 for the treatment of skin infections caused by methicillin-resistant *Staphylococcus aureus* and other Gram-positive pathogens and approved in 2006 for the treatment of endocarditis and bacteraemia caused by *S. aureus*. Daptomycin had also been reported to display strong antibacterial activity against other important pathogens, such as penicillin-resistant *Streptococcus pneumoniae*, coagulase-negative *Staphylococci*, glycopeptide-intermediate-susceptible *S. aureus* and vancomycin-resistant *Enterococci* [166].

Biofilms are groups of bacteria and other organic matter that has colonised/accumulated on a given surface [117]. Bacterial adherence to the surface is the first step in the establishment of biofilm and is affected by various factors, such as the type of microorganism, hydrophobicity, electrical charges of the surface, environmental conditions and the ability of microorganisms to produce extracellular polymers that assist the cells in anchoring to the surface [167]. Anti-adherent activity, which is the ability to inhibit the adherence of pathogenic microorganisms to solid surfaces or infectious sites, has also been reported for biosurfactants, leading to a reduction in hospital infections with no need for drugs or synthetic chemical agents [112]. Meylheuc *et al.* [168] studied a biosurfactant obtained from *P. fluorescens* with inhibitory properties regarding the adherence of *Listeria monocytogenes* to stainless steel and polytetrafluoroethylene surfaces. *C. sphaerica*-derived lunasan inhibited the adherence of *P.aeruginosa, Streptococcus agalactiae* and *S. sanguis* between 80% and 92% at a concentration of 10 mg/mL [95], while the biosurfactant rufisan produced by *C. lipolytica* UCP 0988 demonstrated antimicrobial activities against *S. agalactiae*, *S. mutans*, *S. mutans* NS, *S. mutans* HG, *S. sanguis* 12, *S. oralis* J22 at a concentration above the CMC (0.3%). Moreover, the biosurfactant showed anti-adherent activity against most of the microorganisms tested [137].

Deficiency of lung surfactant, which is a protein-phopholipid complex, is responsible for respiratory failure in premature infants. Gene isolation for protein molecules in this surfactant and cloning in bacteria allow fermentative production for medical applications [108]. Sophorolipids from *C. bombicola* have been studied due to their spermicidal and cytotoxic activities as well as anti-HIV action that can reduce the proliferation of acquired immunodeficiency syndrome (AIDS). Sophorolipids have also been studied as anti-inflammatory agents for patients with immune diseases [108,169]. Iturin is a lipopeptide produced by *B. subtilis* that has demonstrated antifungal activity by affecting the morphology and structure of the cell membrane of yeasts. *In vitro* experiments have demonstrated that surfactin can effectively inactivate the virus that causes herpes as well as the retrovirus and other compact RNA and DNA viruses. The antiviral activity of surfactin has been determined for a broad spectrum of viruses. Moreover, surfactin has been found to exert an effect on insulin absorption in the lungs of laboratory rats [108].

### 11.7. Nanaotechnology

Biosurfactants have been used in nanotechnology and nanoparticle synthesis is emerging as part of green chemistry [118,170]. Nickel oxide (NiO) nano-rods can be produced by water-in-oil microemulsions [171]. In one experiment, two microemulsions were formed with the addition of a nickel chloride solution to a biosurfactant and heptane solution, with the addition of ammonium hydroxide to the same hydrocarbon mixture. The centrifuged microemulsions and ethanol was then used to wash the precipitates and remove the biosurfactant and heptane. The use of biosurfactants is a more ecofriendly approach [119]. Reddy *et al.* [172] found that silver nanoparticle synthesis could be stabilised for two months using surfactin, which is a biodegradable, renewable stabilising agent with low toxicity [3,119]. A biosurfactant produced by *P. Aeruginosa* grown in a low-cost medium has been employed to stabilise silver nanoparticles in the liquid phase [173]. The effect of a rhamnolipid on the electrokinetic and rheological behaviour of nano-zirconia particles has also been analysed, although this is not strictly an environmental application [174]. The biosurfactant increasingly adsorbs to zirconia with the increase in concentration and can serve as an ecofriendly product for the flocculation and dispersion of high solid contents of microparticles. New applications for biosurfactants are being developed in the field of nanotechnology. Future research should focus on the stabilisation of the nanoparticles by biosurfactants before addition during remediation processes [119].

## 12. Future Directions and Concluding Remarks

The biosurfactant industry has demonstrated remarkable growth in recent decades, although the large-scale production of these biomolecules remains a challenge from the economic standpoint. This is mainly due to the enormous difference between the financial investment required and viable industrial production. Thus, the following are the main criteria to be considered for biosurfactant production to become truly viable: (a) type of raw materials; (b) continuous provision of the same composition of ingredients; (c) types of microorganisms; (d) the adequate design of industrial fermentors; (e) financial investments; (f) the target market; (g) purification processes; (h) biosurfactant properties; (i) production conditions, especially the time required for fermentation; (j) adequate production yields; and (k) the processing of recycled products (minimal or able to sell for more than the drop in value).

The target market is of fundamental importance to the implantation of an industrial biosurfactant production project. For cosmetic, medicinal and food products, production is only viable on a small-scale, as the column chromatography methods required to separate molecules are not economical on a large scale. Thus, the use of crude fermentation broths could be a viable solution, especially if the application is in an environmental context, as biosurfactants in such cases do not need to be pure and can be synthesized using a blend of inexpensive carbon sources, which would allow the creation of an economically and environmentally viable technology for bioremediation processes.

According to Hazra *et al.* [140], sophorolipids are offered as sophoron TM from Saraya (Osaka, Japan) and Soliance (Pomacle, France), whereas rhamnolipids are available from Ecover (Boulogne-sur-Mer, France), Jeneil Biosurfactant Inc. (Saukville, Wisconsin, USA) and Rhamnolipid Holdings Inc. (New york, USA). Sophorolipid production costs run from 2 to 5 €/kg. Rhamnolipid production costs US$ 20/kg at a volume of 20 m^3^, but only US$ 5/kg when produced on a scale of 100 m^3^, placing it closer to ethoxylate or alkyl polyglycoside (US$ 1 to 3/kg). The Exxon Company spent more than US$ 10 million in bioremediation studies between 1993 and 1997 after the spillage of petroleum (41 million litres) by the oil tanker Exxon Valdez in Alaska in 1989, leading to the generation of seven patents and making bioremediation second only to enhanced oil recovery within the initial years of use. Distribution in specific biosurfactant areas of the oil industry includes 17 patents for soil and water bioremediation as well as 20 for enhanced oil recovery [140,175].

Although improvements in biosurfactant technology have enabled a 10-to-20-fold increase in the production of these biomolecules, it is likely that further, significant advances (even if of a smaller magnitude) are needed to make this technology commercially viable.

## Figures and Tables

**Table 1:** Main classes of biosurfactants and respective producing microorganisms.

Biosurfactant Class					
Glycolipids	Polymeric Surfactants	Lipopeptides	Fatty Acids	Particulate Surfactant	Phospholipids
Producer microorganisms					
*Acinetobacter calcoaceticus*	*Acinetobacter calcoaceticus* *Acinetobacter calcoaceticus* *Acinetobacter calcoaceticus* *Acinetobacter calcoaceticus* *Bacillus stearothermophilus* *Candida lipolytica* *Candida utilis* *Halomonas eurihalina* *Mycobacterium thermoautotrophium* *Sphingomonas paucimobilis*	*Acinetobacter* sp. *Bacillus licheniformis* *Bacillus pumilus* *Bacillus subtilis* *Candida lipolytica* *Gluconobacter cerinus* *Pseudomonas fluorescens* *Serratia marcescens* *Streptomyces sioyaensis* *Thiobacillus thiooxidans*	*Arthrobacter paraffineus* *Capnocytophaga* sp. *Corynebacterium insidibasseosum* *Corynebacterium lepus* *Nocardia erythropolis* *Penicillium spiculisporum* *Talaramyces trachyspermus*	*Acinetobacter calcoaceticus* *Cyanobacteria* *Pseudomonas marginalis*	*Acinetobacter* sp. *Aspergillus* *Corynebacterium lepus*
*Alcanivorax borkumensis*					
*Arthrobacter paraffineus*					
*Arthrobacter* sp.					
*Candida antartica*					
*Candida apicola*					
*Candida batistae*					
*Candida bogoriensis*					
*Candida bombicola*					
*Candida ishiwadae*					
*Candida lipolytica*					
*Lactobacillus fermentum*					
*Nocardia* sp.					
*Pseudomonas aeruginosa*					
*Pseudomonas* sp.					
*Rhodococcus erythropolis*					
*Rhodotorula glutinus*					
*Rhodotorula graminus*					
*Serratia marcescens*					
*Tsukamurella* sp.					
*Ustilago maydis*					

**Table 2:** Downstream processes for recovery of important biosurfactants and respective advantages.

Process		Biosurfactant Type	Biosurfactant Property Responsible for Separation	Advantages
Batch mode	Acid precipitation	Surfactin	Biosurfactants become insoluble at low pH values	Low cost, efficient in crude biosurfactant recovery
	Organic solvent extraction	Trehalolipids; Sophorolipids; Liposan	Biosurfactants are soluble in organic solvents due to the hydrophobic end	Efficient in crude biosurfactant recovery and partial purification, reusable nature
	Ammonium sulphate precipitation	Emulsan; Biodispersan; Lipopeptides	Salting-out of polymeric or protein-rich biosurfactants	Effective in isolation of certain type of polymeric biosurfactants
Continuous mode	Adsorption to wood-activated carbon	Rhamnolipids; Lipopeptides; Glycolipids; Mannosylerythritol Lipids (MEL)	Biosurfactants are adsorbed to activated carbon and can be desorbed using organic solvents	Highly pure biosurfactants, cheaper, reusability, recovery from continuous culture
	Adsorption to polystyrene resines	Rhamnolipids; Lipopeptides; Glycolipids; MEL	Biosurfactants are adsorbed to polystyrene resins and subsequently desorbed using organic solvents	Highly pure biosurfactants, cheaper, reusability, recovery from continuous culture
	Centrifugation	Glycolipids	Insoluble biosurfactants are precipitated due to centrifugal force	Reusable, effective in crude biosurfactant recovery
	Ion-exchange chromatography	Glycolipids	Charged biosurfactants are attached to ion-exchange resins and can be eluted with buffer	High purity, reusability, fast recovery
	Foam fractionation	Surfactin	Biosurfactant form and partition into foam	Useful in continuous recovery processes, high purity of product
	Ultrafiltration	Glycolipids	Biosurfactants form micelles above their critical micelle concentration (CMC), which are trapped by polymeric membranes	Fast, one-step recovery, high level of purity, reusability

**Table 3:** Applications of biosurfactants for industrial uses.

Industry	Application	Role of Biosurfactants	References
Environment	Bioremediation; Oil spill cleanup operations; Soil remediation and flushing	Emulsification of oils, lowering of interfacial tension, dispersion of oils, solubilisation of oils, wetting, spreading, detergency, foaming, corrosion inhibition in fuel oils and equipment, soil flushing.	[2,8]
Petroleum	Enhanced oil recovery; De-emulsification	Emulsification of oils, lowering of interfacial tension, de-emulsification of oil emulsions, solubilisation of oils, viscosity reduction, dispersion of oils, wetting of solid surfaces, spreading, detergency, foaming, corrosion inhibition in fuel oils and equipment.	[8,120]
Mining	Heavy metal cleanup operations; Soil remediation; Flotation	Wetting and foaming, collectors and frothers, removal of metal ions from aqueous solutions, soil and sediments, heavy metals sequestrants, spreading, corrosion inhibition in oils.	[121]
Food	Emulsification and de-emulsification; Functional ingredient	Solubilisation of flavoured oils, control of consistency, emulsification, wetting agent, spreading, detergency, foaming, thickener.	[4]
Medicine	Microbiological; Pharmaceuticals and therapeutics	Anti-adhesive agents, antifungal agents, antibacterial agents, antiviral agents, vaccines, gene therapy, immunomodulatory molecules.	[20,122,123]
Agriculture	Biocontrol; Fertilisers	Wetting, dispersion, suspension of powdered pesticides and fertilisers, emulsification of pesticide solutions, facilitation of biocontrol mechanisms of microbes, plant pathogen elimination and increased bioavailability of nutrients for beneficial plant-associated microbes.	[124]
Cosmetics	Health and beauty products	Emulsification, foaming agents, solubilisation, wetting agents, cleansers, antimicrobial agents, mediators of enzyme action.	[5]
Cleaning	Washing detergents	Detergents and sanitisers for laundry, wetting, spreading, corrosion inhibition.	[3,5]
Textiles	Preparation of fibres; Dyeing and printing; Finishing of textiles	Wetting, penetration, solubilisation, emulsification, detergency and dispersion, wetting and emulsification in finishing formulations, softening.	[3,103]
Nanotechnology	Synthesis of nanoparticles	Emulsification, stabilisation.	[5,125]

## References

[1] Mao X., Jiang R., Xiao W., Yu J. (2015). Use of surfactants for the remediation of contaminated soils: A review. J. Hazard. Mater..

[2] Silva R.C.F.S., Almeida D.G., Luna J.M., Rufino R.D., Santos V.A., Sarubbo L.A. (2014). Applications of biosurfactants in the petroleum industry and the remediation of oil spills. Int. J. Mol. Sci..

[3] Banat I.M., Franzetti A., Gandolfi I., Bestetti G., Martinotti M.G., Fracchia L., Smyth T.J., Marchant R. (2010). Microbial biosurfactants production, applications. Appl. Microbiol. Biotechnol..

[4] Campos J.M., Stamford T.L.M., Sarubbo L.A., Luna J.M., Rufino R.D., Banat I.M. (2013). Microbial biosurfactants as additives for food industries. Biotechnol. Prog..

[5] Vijayakumar S., Saravanan V. (2015). Biosurfactants-types, sources and applications. Res. J. Microbiol..

[6] Cerqueira V.S., Hollenbach E.B., Maboni F., Vainstein M.H., Camargo F.A., do Carmo M., Peralba R., Bento F.M. (2011). Biodegradation potential of oily sludge by pure and mixed bacterial cultures. Bioresour. Technol..

[7] Rosa C.F.C., Freire D.M.G., Ferraz E.C. (2015). Biosurfactant microfoam: Application in the removal of pollutants from soil. J. Environ. Chem. Eng..

[8] Pacwa-Plociniczak M., Plaza G.A., Piotrowska-Seget Z., Cameotra S.S. (2011). Environmental applications of biosurfactants: Recent advances. Int. J. Mol. Sci..

[9] Makkar R.S., Cameotra S.S., Banat I.M. (2011). Advances in utilization of renewable substrates for biosurfactant production. Appl. Microbiol. Biotechnol..

[10] Deleu M., Paquot M. (2004). From renewable vegetables resources to microorganisms: new trends in surfactants. C. R. Chimie.

[11] Marchant R., Funston S., Uzoigwe C., Rahman P.K.S.M., Banat I.M. (2014). Production of biosurfactants from nonpathogenic bacteria. Biosurfactants.

[12] Chakraborty J., Das S., Das S. (2014). Biosurfactant-based bioremeditaion of toxic metals. Microbial Biodegradation and Bioremediation.

[13] Shepherd R., Rockey J., Shutherland I.W., Roller S. (1995). Novel bioemulsifiers from microorganisms for use in foods. J. Biotechnol..

[14] Bognolo G. (1999). Biossurfactants as emulsifying agents for hydrocarbons. Coll. Surf. A Physicochem. Eng. Asp..

[15] Pattanath K.M., Rahman K.S., Gakpe E. (2008). Production, characterization and applications of biosurfactants—Review. Biotechnology.

[16] Chrzanowski L., Ławniczak L., Czaczyk K. (2012). Why do microorganisms produce rhamnolipids?. World J. Microbiol. Biotechnol..

[17] Abdel-Mawgoud A.M., Lépine F., Deziel E. (2010). Rhamnolipids: Diversity of structures, microbial origins and roles. Appl. Microbiol. Biotechnol..

[18] Hitsatsuka K., Nakahara T., Sano N., Yamada K. (1971). Formation of a rhamnolipid by *Pseudomonas aeruginosa* and its function in hydrocarbon fermentation. Agric. Biol. Chem..

[19] Nitschke M., Costa S.G.V.A.O., Contiero J. (2011). Rhamnolipids and PHAs: Recent reports on *Pseudomonas*-derived molecules of increasing industrial interest. Process Biochem..

[20] Ławniczak L., Marecik R., Chrzanowski L. (2013). Contributions of biosurfactants to natural or induced bioremediation. Appl. Microbiol. Biotechnol..

[21] Chrzanowski L., Dziadas M., Ławniczak L., Cyplik P., Białas W., Szulc A., Lisiecki P., Jelen H. (2012). Biodegradation of rhamnolipids in liquid cultures: Effect of biosurfactant dissipation on diesel fuel/B20 blend biodegradation efficiency and bacterial community composition. Bioresour. Technol..

[22] Szulc A., Ambrozewicz D., Sydow M., Ławniczak L., Piotrowska-Cyplik A., Marecik R., Chrzanowski L. (2014). The influence of bioaugmentation and biosurfactant addition on bioremediation efficiency of diesel-oil contaminated soil: Feasibility during field studies. J. Environ. Manag..

[23] Marecik M., Wojtera-Kwiczor J., Ławniczak L., Cyplik P., Szulc A., Piotrowska-Cyplik A., Chrzanowski L. (2012). Rhamnolipids increase the phytotoxicity of diesel oil towards four common plant species in a terrestrial environment. Water Air Soil Pollut..

[24] Chrzanowski L., Wick L.Y., Meulenkamp R., Kaestner M., Heipieper H.J. (2009). Rhamnolipid biosurfactants decrease the toxicity of chlorinated phenols to *Pseudomonas putida* DOT-T1E. Lett. Appl. Microbiol..

[25] Chrzanowski L., Owsianiak M., Szulc A., Marecik R., Piotrowska-Cyplik A., Olejnik-Schmidt A.K., Staniewski J., Lisiecki P., Ciesielczyk F., Jesionowski T. (2011). Interactions between rhamnolipid biosurfactants and toxic chlorinated phenols enhance biodegradation of a model hydrocarbon-rich effluent. Int. Biodeterior. Biodegrad..

[26] Cortés-Sánchez A.J., Sánchez H.H., Jaramillo-Flores M.E. (2013). Biological activity of glycolipids produced by microorganisms: New trends and possible therapeutic alternatives. Microbiol. Res..

[27] Daverey A., Pakshirajan K. (2009). Production, characterization, and properties of sophorolipids from the yeast *Candida bombicola* using a low-cost fermentative medium. Appl. Biochem. Biotechnol..

[28] Gautam K.K., Tyagi V.K. (2006). Microbial Surfactants: A review. J. Oleo Sci..

[29] Hu Y., Ju L.K. (2001). Purification of lactonic sophorolipids by crystallization. J. Biotechnol..

[30] Díaz De Rienzo M.A.D., Banat I.M., Dolman B., Winterburn J., Martin P.J. (2015). Sophorolipid biosurfactants: Possible uses as antibacterial and antibiofilm agent. New Biotechnol..

[31] Lang S. (2002). Biological amphiphiles (microbial biosurfactants). Curr. Opin. Coll. Interface Sci..

[32] Hatha A.A.M., Edward G., Rahman K.S.M.P. (2007). Microbial biosurfactants-review. J. Mar. Atmos. Res..

[33] Chakrabarti S. (2012). Bacterial Biosurfactant: Characterization, Antimicrobial and Metal Remediation Properties. Ph.D. Thesis.

[34] Barros F.F.C., Quadros C.P., Maróstica M.R., Pastore G.M. (2007). Surfactina: Propriedades químicas, tecnológicas e funcionais para aplicações em alimentos. Quím. Nova.

[35] Velikonja J., Kosaric N., Kosaric N., Sukan F.V. (1993). Biosurfactants in food applications. Biosurfactants: Production, Properties, Applications.

[36] Cirigliano M.C., Carman G.M. (1985). Purification and characterization of liposan, a bioemulsifier from *Candida lipolytica*. Appl. Microbiol. Biotechnol..

[37] Desai J.D., Banat I.M. (1997). Microbial production of surfactants and their commercial potential. Microbiol. Mol. Biol. Rev..

[38] Bhardwaj G., Cameotra S.S., Chopra H.K. (2013). Biosurfactants from Fungi: A Review. Pet. Environ. Biotechnol..

[39] Cooper D.G., Paddock D.A. (1984). Production of a biosurfactant from *Torulopsis bombicola*. Appl. Environ. Microbiol..

[40] Kim S.Y., Oh D.K., Lee K.H., Kim J.H. (1997). Effect of soybean oil and glucose on sophorose lipid fermentation by *Torulopsis bombicola* in continuous culture. Appl. Microbiol. Biotechnol..

[41] Casas J.A., de Lara S.G., Garcia-Ochoa F. (1997). Optimization of a synthetic medium for *Candida bombicola* growth using factorial design of experiments. Enzyme Microb. Technol..

[42] Sarubbo L.A., Farias C.B.B., Campos-Takaki G.M. (2007). Co-utilization of canola oil and glucose on the production of a surfactant by *Candida lipolytica*. Curr. Microbiol..

[43] Rufino R.D., Sarubbo L.A., Campos-Takaki G.M. (2007). Enhancement of stability of biosurfactant produced by *Candida lipolytica* using industrial residue as substrate. World J. Microbiol. Biotechnol..

[44] Sarubbo L.A., Porto A.L.F., Campos-Takaki G.M. (2001). Bioemulsifier production in batch culture using glucose as carbon source by *Candida lipolytica*. Appl. Biochem. Biotechnol..

[45] Bednarski W., Adamczak M., Tomasik J., Plaszvzyk M. (2004). Application of oil refinery waste in the biosynthesis of glycolipids by yeast. Bioresour. Technol..

[46] Kitamoto D., Ikegami T., Suzuki G.T., Sasaki A., Takeyama Y., Idemoto Y., Koura N., Yanagishita H. (2001). Microbial conversion of n-alkanes into glycolipid biosurfactants, mannosylerythritol lipids by *Pseudozyma* (*Candida antartica*). Biotechnol. Lett..

[47] Amezcua-Veja C., Poggi-Varaldo H.M., Esparza-Garcia F., Rodriguez-Vazquez R. (2006). Effect of culture conditions on fatty acids composition of a biosurfactant produced by *Candida ingens* and of surface tension of culture media. Bioresour. Technol..

[48] Robert M., Mercadé M.E., Bosch M.P., Parra J.L., Espiny M.J., Manresa M.A., Guinea J. (1989). Effect of the carbon source on biosurfactant production by *Pseudomonas aeruginosa* 44T1. Biotechnol. Lett..

[49] Santa Anna I.M., Sebastian G.V., Pereira N., Alves T.L.M., Menezes E.P., Freire D.M.G. (2002). Production of biosurfactant from a new and promissing strain of *Pseudomonas aeruginosa* PA1. Appl. Biochem. Biotechnol..

[50] Mulligan C.N., Gibbs B.F. (1989). Correlation of nitrogen metabolism with biosurfactant production by *Pseudomonas aeruginosa*. Appl. Environ. Microbiol..

[51] Deshpande M., Daniels L. (1995). Evaluation of sophorolipid biosurfactant production by *Candida bombicola* using animal fat. Bioresour. Technol..

[52] Kim H.S., Yoon B.D., Choung D.H., Oh H.M., Katsuragi T. (1999). Characterization of a biosurfactant mannosylerythritol lipid produced from *Candida* sp. SY 16. Appl. Microbiol. Biotechnol..

[53] Rufino R.D., Sarubbo L.A., Benicio B.N., Campos-Takaki G.M. (2008). Experimental design for the production of tensio-active agent by *Candida lipolytica*. J. Ind. Microbiol. Biotechnol..

[54] Sarubbo L.A., Luna J.M., Campos-Takaki G.M. (2006). Production and stability studies of the bioemulsifier obtained from a new strain of *Candida glabrata* UCP 1002. Electr. J. Biotechnol..

[55] Cirigliano M.C., Carman G.M. (1984). Isolation of a bioemulsifier from *Candida lipolytica*. Appl. Environ. Microbiol..

[56] Konishi M., Fukuoka T., Morita T., Imura T., Kitamoto D. (2008). Production of new types of sophorolipids by *Candida batistae*. J. Oleo Sci..

[57] Kiran G.S., Hema T.A., Gandhimathi R., Selvin J., Thomas T.A. (2009). Optimization and production of a biosurfactant from the sponge-associated marine fungus *Aspergillus ustus* MSF3. Coll. Surf. B Biointerfaces.

[58] Thaniyavarn J., Chianguthai T., Sangvanich P., Roongsawang N., Washio K., Morikawa M., Thaniyavarn S. (2008). Production of sophorolipid biosurfactant by *Pichia anomala*. Biosci. Biotechnol. Biochem..

[59] Cavalero D.A., Cooper D.G. (2003). The effect of medium composition on the structure and physical state of sophorolipids produced by *Candida bombicola* ATCC 22214. J. Biotechnol..

[60] Felse P.A., Shah V., Chan J., Rao K.J., Gross R.A. (2007). Sophorolipid biosynthesis by *Candida bombicola* from industrial fatty acid residues. Enzyme Microb. Technol..

[61] Silva S.N.R.L., Farias C.B.B., Rufino R.D., Luna J.M., Sarubbo L.A. (2010). Glycerol as substrate for the production of biosurfactant by *Pseudomonas aeruginosa* UCP0992. Coll. Surf. B Biointerfaces.

[62] Oliveira F.J.S., Vazquez L., de Campos N.P., de Franca F.P. (2009). Production of rhamnolipids by a *Pseudomonas alcaligenes* strain. Process Biochem..

[63] Cunha C.D., Rosario M., Rosado A.S., Leite G.F. (2004). *Serratia* sp. SVGG16: A promising biosurfactant producer isolated from tropical soil during growth with ethanol-blended gasoline. Process Biochem..

[64] Weber L., Doge C., Haufe G., Hommel R., Kleber H.-P. (1992). Oxygenation of hexadecane in the biosynthesis of cyclic glycolipids in *Torulopsis apicola*. Biocatal.

[65] Haritash A.K., Kaushik C.P. (2009). Biodegradation aspects of Polycyclic Aromatic Hydrocarbons (PAHs): A review. J. Hazard. Mater..

[66] Hommel R.K., Huse K. (1993). Regulation of sophorose lipid production by *Candida* (*Torulopsis*) *apicola*. Biotechnol. Lett..

[67] Tokumoto Y., Nomura N., Uchiyama H., Imura T., Morita T., Fukuoka T., Kitamoto D. (2009). Structural characterization and surface-active properties of a succinoyl trehalose lipid produce by *Rhodococcus* sp. SD-74. J. Oleo Sci..

[68] Syldatk C., Wagner F., Kosaric N., Cairns W.L., Gray N.C.C. (1987). Production of biosurfactants. Biosurfactants and Biotechnology.

[69] Tan H.M. (2000). Biosurfactants and their roles in bioremediation. Cheong Jit Kong.

[70] Banat I.M., Satpute S.K., Cameotra S.S., Patil R., Nyayanit N. (2014). Cost effective technologies and renewable substrates for biosurfactants’ production. Front. Microbiol..

[71] Makkar R.S., Cameotra S.S. (2002). An update on the use of uncoventional substrates for biosurfactant production and their new applications. Appl. Micobiol. Biotechnol..

[72] Batista R.M., Rufino R.D., Luna J.M., Souza J.E.G., Sarubbo L.A. (2010). Effect of Medium components on the production of a biosurfactant from *Candida tropicalis* applied to the removal of hydrophobic contaminants in soil. Water Environ. Res..

[73] Mercade M.E., Manresa A., Robert M., Espuny M.J., Deandres C., Guinea J. (1993). Olive oil mill effluent (OOME). New substrat for biosurfactant production. Bioresour. Technol..

[74] Fox S.I., Bala G.A. (2000). Production of surfactant from *Bacillus subtilis* ATTCC 21332 using potato substrates. Bioresour. Technol..

[75] Thompson D.N., Fox S.L., Bala G.A. (2000). Biosurfactants from potato process effluents. Appl. Biochem. Biotechnol..

[76] Maneerat S. (2005). Production of biosurfactants using substrates from renewable-resources. Songklanakarin J. Sci. Technol..

[77] Santos D.K.F., Brandão Y.B., Rufino R.D., Luna J.M., Salgueiro A.A., Santos V.A., Sarubbo L.A. (2014). Optimization of cultural conditions for biosurfactant production from *Candida lipolytica*. Biocatal. Agric. Biotechnol..

[78] Santos D.K.F., Rufino R.D., Luna J.M., Santos V.A., Salgueiro A.A., Sarubbo L.A. (2013). Synthesis and evaluation of biosurfactant produced by *Candida lipolytica* using animal fat and corn steep liquor. J. Pet. Sci. Eng..

[79] Gusmão C.A.B., Rufino R.D., Sarubbo L.A. (2010). Laboratory production and characterization of a new biosurfactant from *Candida glabrata* UCP 1002 cultivated in vegetable fat waste applied to the removal of hydrophobic contaminant. World J. Microbiol. Biotechnol..

[80] Alcântara R., Amores J., Canoira L., Fidalgo E., Franco M.J., Navarro A. (2000). Catalytic production of biodiesel from soy-hean oil, used frying oil and tallow. Biomass Bioenergy.

[81] Cvengros Z., Cvengrosova Z. (2004). Used fruing oils and fats and their utilization in the production of methyl ester of higher fatty acids. Biomass Bioenergy.

[82] Haba E., Espuny M.J., Busquets M., Manresa A. (2000). Screening and production of rhamnolipids *Pseudomonas aeruginosa* 47T2 NCIB 40044 from waste flying oils. J. Appl. Microbiol..

[83] Benincasa M., Contiero J., Manresa M.A., Moraes I.O. (2002). Rhamnolipid production by *Pseudomonas aeruginosa* LBI growing on soapstock as the sole carbon source. J. Food Eng..

[84] Shabtai Y. (1990). Production of exopolysaccharides by *Acinetobacter* strains in a controlled fed-batch fermentation process using soap stock oil (SSO) as carbon source. Int. J. Biol. Macromol..

[85] Ghurye G.L., Vipulananda C., Wilson R.C. (1994). A practical approach to biosurfactant production using nonaseptic fermantation of mixed cultures. Biotechnol. Bioeng..

[86] Kalogiannis S., Iakovidou G., Liakopoulou-Kyriakides M., Kyriakidis D.A., Skaracis G.N. (2003). Optimization of xanthan gum production by *Xanthomonas campestris* grown in molasses. Process Biochem..

[87] Lazaridou A., Roukas T., Biliaderis C.G., Vaikousi H. (2002). Characterization of pullulan produced from beet molasses by *Aureobasidium pullulans* in a stirred tank reactor under varying agitation. Enzyme Microbiol. Technol..

[88] Makkar R.S., Cameotra S.S. (1997). Utilization of molasses for biosurfactant production by two *Bacillus* strains at thermophilic conditions. J. Am. Oil Chem. Soc..

[89] Sudhakar-Babu P., Vaidya A.N., Bal A.S., Kapur R., Juwarka A., Khanna P. (1996). Kinetics of biosurfactants production by *Pseudomonas aeruginosa* strain from industrial wastes. Biotechnol. Lett..

[90] Luna J.M., Rufino R.D., Albuquerque C.D.C., Sarubbo L.A., Campos-Takaki G.M. (2011). Economic optimized medium for tenso-active agent production by *Candida sphaerica* UCP 0995 and application in the removal of hydrophobic contaminant from sand. Int. J. Mol. Sci..

[91] Rufino R.D., Luna J.M., Rodrigues G.I.B., Campos-Takaki G.M., Sarubbo L.A., Ferreira S.R.M. (2011). Application of a yeast biosurfactant in the removal of heavy metals and hydrophobic contaminant in a soil used as slurry barrier. Appl. Environ. Soil Sci..

[92] Sobrinho H.B.S., Rufino R.D., Luna J.M., Salgueiro A.A., Campos-Takaki G.M., Leite L.F.C., Sarubbo L.A. (2008). Utilization of two agroindustrial by-products for the production of a surfactant by *Candida sphaerica* UCP 0995. Process Biochem..

[93] Nitschke M., Ferraz C., Pastore G.M. (2004). Selection of microorganisms for biosurfactant production using agroindustrial wastes. Braz. J. Microbiol..

[94] Luna J.M., Rufino R.D., Sarubbo L.A., Campos-Takaki G.M. (2013). Characterisation, surface properties and biological activity of a biosurfactant produced from industrial waste by *Candida sphaerica* UCP 0995 for application in the petroleum industry. Coll. Surf. B Biointerfaces.

[95] Luna J.M., Rufino R.D., Sarubbo L.A., Rodrigues L.R.M., Teixeira J.A.C., Campos-Takaki G.M. (2011). Evaluation antimicrobial and antiadhesive properties of the biosurfactant Lunasan produced by *Candida sphaerica* UCP 0995. Curr. Microbiol..

[96] Sarubbo L.A., Marçal M.C., Neves M.L.C., Porto A.L.F., Campos-Takaki G.M. (1999). The use of babassu oil as substrate to produce bioemulsifiers by *Candida lipolytica*. Can. J. Microbiol..

[97] Raza Z.A., Khan M.S., Khalid Z.M., Rehman A. (2006). Production kinetics and tensioactive characteristics of biosurfactant from a *Pseudomonas aeruginosa* mutant grown on waste frying oils. Biotechnol. Lett..

[98] Raza Z.A., Rehman A., Khan M.S., Khalid Z.M. (2007). Improved production of biosurfactant by a *Pseudomonas aeruginosa* mutant using vegetable oil refinery wastes. Biodegradation.

[99] Joshi S., Bharucha C., Jha S., Yadav S., Nerurkar A., Desai A.J. (2007). Biosurfactant production using molasses and whey under thermophilic conditions. Bioresour. Technol..

[100] Dubey K.V., Juwarkar A.A. (2001). Distillery and curd whey wastes as viable alternative sources for biosurfactant production. World J. Microbiol. Biotechnol..

[101] Dubey K.V., Juwarkar A.A., Singh S.K. (2005). Adsorption-desorption process using woodbased activated carbon for recovery of biosurfactant from fermented distillery wastewater. Biotechnol. Prog..

[102] Rodrigues L.R., Teixeira J.A., Oliveira R. (2006). Low-cost fermentative medium for biosurfactant production by probiotic bacteria. Biochem. Eng. J..

[103] Helmy Q., Kardena E., Funamizu N., Wisjnuprapto W. (2011). Strategies toward commercial scale of biosurfactant production as potential substitute for it’s chemically counterparts. Int. J. Biotechnol..

[104] Akhtar M., Lentz M.J., Banchette R.A., Kirk T.K. (1997). Corn steep liquor lowers the amount of inoculum for biopulping. TAPPI J..

[105] Cardinal E.V., Hedrick L.R. (1948). Microbiological assay of corn steep liquor for amino acid content. J. Biol. Chem..

[106] Luna J.M., Rufino R.D., Jara A.M.T., Brasileiro P.P.F., Sarubbo L.A. (2015). Environmental applications of the biosurfactant produced by *Candida sphaerica* cultivated in low-cost substrates. Coll. Surf. A Physicochem. Eng. Asp..

[107] Silva N.M.P.R., Rufino R.D., Luna J.M., Santos V.A., Sarubbo L.A. (2014). Screening of *Pseudomonas* species for biosurfactant production using low-cost substrates. Biocatal. Agric. Biotechnol..

[108] Muthusamy K., Gopalakrishnan S., Ravi T.K., Sivachidambaram P. (2008). Biosurfactants: Properties, commercial production and application. Curr. Sci..

[109] Marchant R., Banat I.M. (2012). Biosurfactants: A sustainable replacement for chemical surfactants?. Biotechnol. Lett..

[110] Winterburn J.B., Russell A.B., Martin P.J. (2011). Integrated recirculating foam fractionation for the continuous recovery of biosurfactant from fermenters. Biochem. Eng. J..

[111] Rangarajan V., Sem R. (2013). An inexpensive strategy for facilitated recovery of metals and fermentation products by foam fractionation process. Coll. Surf. B Biointerfaces.

[112] Rodrigues L.R., Banat I.M., Teixeira J.A., Oliveira R. (2006). Biosurfactants: Potential applications in medicine. J. Antimicrob. Chemother..

[113] Sobrinho H.B.S., Luna J.M., Rufino R.D., Porto A.L.F., Sarubbo L.A. (2013). Assessment of toxicity of a biosurfactant from *Candida sphaerica* UCP 0995 cultivated with industrial residues in a bioreactor. Electron. J. Biotechnol..

[114] US Energy Information Administration Annual Energy Review, 2010. http://large.stanford.edu/courses/2012/ph241/druzgalski2/docs/aer.pdf.

[115] Summers D. Enhancing Oil Recovery: A Look at Stripper Wells. http//www.oilprice.com.

[116] Austad T., Taugbøl K. (1995). Chemical flooding of oil reservoirs 1. Low tension polymer flood using a polymer gradient in the three-phase region. Coll. Surf. A Phys. Eng. Asp..

[117] West C.C., Harwell J.H. (1992). Surfactant and subsurface remediation. Environ. Sci. Technol..

[118] Sabatini D.A., Harwell J.H., Knox R.C., Brusseau M.L., Sabatini D.A., Gierke J.S., Annable M.D. (1999). Surfactant selection criteria for enhanced subsurface remediation. Innovative Subsurface Remediation.

[119] Sabatini D.A., Knox R.C., Harwell J.H., Wu B. (2000). Integrated design of surfactant enhanced DNAPL remediation: Efficient super solubilization and gradient systems. J. Contam. Hydrol..

[120] Souza E.C., Vessoni-Penna T.C., Souza Oliveira R.P. (2014). Biosurfactant-enhanced hydrocarbon bioremediation: An overview. Int. Biodeterior. Biodegrad..

[121] Sarubbo L.A., Rocha R.B., Luna J.M., Rufino R.D., Santos V.A., Banat I.M. (2015). Some aspects of heavy metals contamination remediation and role of biosurfactants. Chem. Ecol..

[122] Mnif I., Ghribi D. (2015). Microbial derived surface active compounds: Properties and screening concept. World J. Microbiol. Biotechnol..

[123] Banat I.M., De Rienzo M.A.D., Quinn G.A. (2014). Microbial biofilms: Biosurfactants as antibiofilm agents. Appl. Microbiol. Biotechnol..

[124] Sachdev D.P., Cameotra S.S. (2013). Biosurfactants in agriculture. Appl. Microbiol. Biotechnol..

[125] Mulligan C.N. (2009). Recent advances in the environmental applications of biosurfactants. Curr. Opin. Coll. Interface Sci..

[126] Environmental Protection Agency (EPA), U.S. (2001). Treatment Technologies for Site Cleanup: Annual Status Report.

[127] Satpute S.K., Banat I.M., Dhakephalkar P.K., Banpurkar A.G., Chopade B.A. (2010). Biosurfactants, bioemulsifiers and exopolysaccharides from marine microorganisms. Biotechnol. Adv..

[128] Shavandi M., Mohebali G., Haddadi A., Shakarami H., Nuhi A. (2011). Emulsification potential of a newly isolated biosurfactantproducing bacterium, *Rhodococcus* sp. strain TA6. Coll. Surf. B Biointerfaces.

[129] Kuyukina M.S., Ivshina I.B., Makarov S.O., Litvinenko L.V., Cunningham C.J., Philip J.C. (2005). Effect of biosurfactants on crude oil desorption and mobilization in a soil system. Environ. Int..

[130] Aparna A., Srinikethan G., Hedge S. Effect of addition of biosurfactant produced by *Pseudomonas* ssp. on biodegradation of crude oil. International Proceedings of Chemical, Biological & Environmental Engineering, Proceedings of the 2nd International Proceedings of Chemical.

[131] Franzetti A., Caredda P., Ruggeri C., La Colla P., Tamburini E., Papacchini M., Bestetti G. (2009). Potential applications of surface active compounds by *Gordonia* sp. strain BS29 in soil remediation technologies. Chemosphere.

[132] Bai G., Brusseau M.L., Miller R.M. (1997). Biosurfactant-enhanced removal of residual hydrocarbon from soil. J. Contam. Hydrol..

[133] Silva R.C.F.S., Rufino R.D., Luna J.M., Farias C.B.B., Filho H.J.B., Santos V.A., Sarubbo L.A. (2013). Enhancement of biosurfactant production from *Pseudomonas cepacia* CCT6659 through optimisation of nutritional parameters using Response Surface Methodology. Tenside Surf. Det..

[134] Chaprão M.J., Ferreira I.N.S., Correa P.F., Rufino R.D., Luna J.M., Silva E.J., Sarubbo L.A. (2015). Application of bacterial and yeast biosurfactants for enhanced removal and biodegradation of motor oil from contaminated sand. Electron. J. Biotechnol..

[135] Rufino R.D., Luna J.M., Campos Takaki G.M., Sarubbo L.A. (2014). Characterization and properties of the biosurfactant produced by *Candida lipolytica* UCP 0988. Electron. J. Biotechnol..

[136] Rufino R.D., Luna J.M., Marinho P.H.C., Farias C.B.B., Ferreira S.R.M., Sarubbo L.A. (2013). Removal of petroleum derivative adsorbed to soil by biosurfactant Rufisan produced by *Candida lipolytica*. J. Pet. Sci. Eng..

[137] Rufino R.D., Luna J.M., Sarubbo L.A., Rodrigues L.R.M., Teixeira J.A.C., Campos-Takaki G.M. (2011). Antimicrobial and anti-adhesive potential of a biosurfactant Rufisan produced by *Candida lipolytica* UCP 0988. Coll. Surf. B Biointerfaces.

[138] Mulligan C.N., Wang S. (2004). Remediation of a heavy metal contaminated soil by a rhamnolipid foam. Geoenvironmental Engineering: Integrated Management of Groundwater and Contaminated Land.

[139] Singh A., Van-Hamme J.D., Ward O.P. (2007). Surfactants in microbiology and biotechnology: Part 2. Application aspects. Biotechnol. Adv..

[140] Hazra C., Kundu D., Chaudhari A., Satyanarayana T., Johri B.N., Prakash A. (2012). Biosurfactant-assisted bioaugmentation in bioremediation. Microorganisms in Environmental Management: Microbes and Environment.

[141] Ochoa-Loza F.J., Artiola J.F., Maier R.M. (2001). Stability constants for the complexation of various metals with a rhamnolipid biosurfactant. J. Environ. Qual..

[142] Swarnkar V., Agrawal N., Tomar R. (2012). Sorption of chromate and arsenate bysurfactant modified erionite (E-SMZ). J. Dispers. Sci. Technol..

[143] Hashim M.A., Mukhopadhyay S., Sahu J.N., Sengupta B. (2011). Remediation technologies for heavy metal contaminated groundwater. J. Environ. Manag..

[144] Asçi Y., Nurbas M., Açikel Y. (2008). A comparative study for the sorption of Cd(II) by soils with different clay contents and mineralogy and the recovery of Cd(II) using rhamnolipid biosurfactant. J. Hazard. Mater..

[145] Nash J., Traver R.P., Downey D.C. (1987). Surfactant enhanced *in situ* soil washing.

[146] Herman D.C., Artiola J.F., Miller R.M. (1995). Removal of cadmium, lead and zinc from soil by a rhamnolipid biosurfactant. Environ. Sci. Technol..

[147] Mulligan C.N., Yong R.N., Gibbs B.F. (1999). Metal removal from contaminated soil and sediments by the biosurfactant surfactin. Environ. Sci. Technol..

[148] Ochoa-Loza F.J., Noordman W.H., Jannsen D.B., Brusseau M.L., Maier R.M. (2007). Effect of clays, metal oxides, and organic matter on rhaminolipid biosurfactant sorpition by soil. Chemosphere.

[149] Das P., Mukherjee S., Sen R. (2009). Antiadhesive action of a marine microbial surfactant. Coll. Surf. B Biointerfaces.

[150] Wen J., Stacey S.P., Mclaughlin M.J., Kirby J.K. (2009). Biodegradation of rhamnolipid, EDTA and citric acid in cadmium and zinc contaminated soils. Soil Biol. Biochem..

[151] Kitamoto D., Isoda H., Nakahara T. (2002). Functions and potential applications of glycolipid biosurfactants: From energy-saving materials to gene delivery carriers. J. Biosci. Bioeng..

[152] Nitschke M., Pastore G.M. (2002). Biossurfactantes: Propriedades e aplicações. Química Nova.

[153] Slizovskiy I.B., Kelsey J.W., Hatzinger P.B. (2011). Surfactant-facilitated remediationof metal-contaminated soils: Efficacy and toxicological consequences to earth worms. Environ. Toxicol. Chem..

[154] Almeida C.M.R., Dias A.C., Mucha A.P., Bordalo A.A., Vasconcelos M.T.S.D. (2009). Influence of surfactants on the Cu phytoremediation potential of a saltmarsh plant. Chemosphere.

[155] Maity J.P., Huang Y.M., Fan C.-W., Chen C.-C., Li C.-Y., Hsu C.-M., Chang Y.-F., Wu C.-I., Chen C.-Y., Jean J.-S. (2013). Evaluation of remediation process with soapberry derived saponin for removal of heavy metals from contaminated soils in Hai-Pu Taiwan. J. Environ. Sci..

[156] Ozturk S., Kaya T., Aslim B., Tan S. (2012). Removal and reduction of chromium by *Pseudomonas* spp. and their correlation to rhamnolipid production. J. Hazard. Mater..

[157] Albuquerque C.F., Luna-Finkler C.L., Rufino R.D., Luna J.M., Menezes C.T.B., Santos V.A., Sarubbo L.A. (2012). Evaluation of biosurfactants for removal of heavy metal ions from aqueous effluent using flotation techniques. Int. Rev. Chem. Eng..

[158] Menezes C.T.B., Barros E.C., Rufino R.D., Luna J.M., Sarubbo L.A. (2011). Replacing synthetic with microbial surfactants as collectors in the treatment of aqueous effluent produced by acid mine drainage using the dissolved air flotation technique. Appl. Biochem. Biotechnol..

[159] Campos J.M., Stamford T.L.M., Sarubbo L.A. (2014). Production of a Bioemulsifier with Potential Application in the Food Industry. Appl. Biochem. Biotechnol..

[160] Campos J.M., Stamford T.L.M., Rufino R.D., Luna J.M., Stamford T.C.M., Sarubbo L.A. (2015). Formulation of mayonnaise with the addition of a bioemulsifier isolated from *Candida utilis*. Toxicol. Rep..

[161] Torabizadeh H., Shojaosadati S.A., Tehrani H.A. (1996). Preparation and characterization of bioemulsifier from *Saccharomyces cerevisiae*. Lebensm. Wiss. Technol..

[162] Abalos A., Pinazo A., Infante M.R., Casals M., García F., Manresa A. (2001). Physicochemical and antimicrobial properties of new rhamnolipids produced by *Pseudomonas aeruginosa* AT10 from soybean oil refinery wastes. Langmuir.

[163] Joshi-Navare K., Prabhune A. (2013). A biosurfactant sophorolipid acts in synergy with antibiotics to enhance their efficiency. BioMed. Res. Int..

[164] Fracchia L., Banat J.J., Cavallo M., Ceresa C., Banat I.M. (2015). Potential therapeutic applications of microbial surface-active compounds. Bioengineering.

[165] Robbel L., Marahiel M.A. (2010). Daptomycin, a bacterial lipopeptide synthesized by a nonribosomal machinery. J. Biol. Chem..

[166] Tally F.P., Zeckel M., Wasilewski M.M. (1999). Daptomycin, A novel agent for Gram-positive infections. Expert Opin. Investig. Drug.

[167] Zottola E.A. (1994). Microbial attachment and biofilm formation: A new problem for the food industry?. Food Technol..

[168] Meylheuc T., Van Oss C.J., Bellon-Fontaine M.N. (2001). Adsorption of biosurfactants on solid surfaces and consequences regarding the bioahesion of *Listeria monocytogenes* LO28. J. Appl. Microbiol..

[169] Kitamoto D., Morita T., Fukuoka T., Konishi M., Imura T. (2009). Self-assembling properties of glycolipid biosurfactants and their potential applications. Curr. Opin. Coll. Interface Sci..

[170] Kiran G.S., Sabu A., Selvin J. (2010). Synthesis of silver nanoparticles by glycolipid biosurfactant produced from marine *Brevibacterium casei* MSA19. J. Biotechnol..

[171] Palanisamy P. (2008). Biosurfactant mediated synthesis of NiO nanorods. Mater. Lett..

[172] Reddy A.S., Chen C.-Y., Baker S.C., Chen C.-C., Jean J.-S., Fan C.-W., Chen H.-R., Wang J.-C. (2009). Synthesis of silver nanoparticles using surfactin: A biosurfactant stabilizing agent. Mater Lett..

[173] Farias C.B.B., Silva A.F., Rufino R.D., Luna J.M., Souza J.E., Sarubbo L.A. (2014). Synthesis of silver nanoparticles using a biosurfactant produced in low-cost medium as stabilizing agent. Electron. J. Biotechnol..

[174] Biswas M., Raichur A.M. (2008). Electrokinetic and rheological properties of nano zirconia in the presence of rhamnolipid biosurfactant. J. Am. Ceram. Soc..

[175] Santos H.F., Carmo F.L., Paes J.E.S., Rosado A.S., Peixoto R.S. (2011). Bioremediation of mangroves impacted by petroleum. Water Air Soil Pollut..

